# The role of diet and nutrition related indicators in biliary diseases: an umbrella review of systematic review and meta-analysis

**DOI:** 10.1186/s12986-022-00677-1

**Published:** 2022-07-30

**Authors:** Yaoqun Wang, Jiong Lu, Ningyuan Wen, Guilin Nie, Dingzhong Peng, Xianze Xiong, Nansheng Cheng, Bei Li

**Affiliations:** Division of Biliary Surgery, Department of General Surgery, West China Hospital of Sichuan University, No. 37 Guo Xue Xiang, Chengdu, 610041 Sichuan Province China

**Keywords:** Diet, Biliary diseases, Umbrella review, Meta-analysis, Systematic review

## Abstract

**Background:**

Diet and nutrition, as a modifiable risk factor, have been demonstrated to play a significant role in the etiology of biliary diseases, whereas few comprehensive studies have been able to evaluate the strength and quality of these evidence. This umbrella review aims to evaluate the evidence pertaining risk factors for biliary diseases in terms of diet and nutrition-related indicators.

**Methods:**

An umbrella review method was adopted: evidence from observational studies up to 22 November 2021 were identified using PubMed, Web of Science, the Cochrane database, as well as manual screening. Eligible systematic reviews and meta-analyses were screened according to inclusion and exclusion criteria. The inclusion criteria were: (1) meta analysis or systematic review; (2) The theme of the study is the relationship between diet or nutrition and biliary tract diseases; (3) Summarized and reported OR, RR or HR values and corresponding 95% CI; (4) No restrictions on the use of participants and languages; (5) Only extract the data of biliary tract diseases from multiple health outcomes; (6) Only the most recent studies on the same subject were included. This study had been registered at PROSPERO (CRD42021293908). For each eligible systematic review and meta-analysis, we extracted the data of general characteristics and the main findings. The methodological quality of the meta-analyses included in our study were assessed by AMSTAR2 and the quality of evidence was evaluated by the GRADE.

**Results:**

A total of 323 articles were searched, among which 24 articles with 83 unique outcomes were identified as eligible. 35 of these outcomes were downgraded in GRADE evaluation as they reported heterogeneity. In short, among 83 unique outcomes, 5 were rated as moderate, 16 as low, and the rest as very low. For the prevention of biliary tract diseases, emphasis should be placed on appropriately increasing the intake of fruits, vegetables, coffee and tea, and reducing the intake of alcohol, raw fish and foods with high nitrate. Meanwhile, weight, blood sugar and lipid levels should be controlled, and diabetes should be actively prevented and treated. Drinking is not recommended to prevent gallstones, although studies have shown that it may reduce the risk of cholecystolithiasis.

**Conclusions:**

Our study summarizes the current multifaceted evidence on the relationship between dietary and nutritional indicators and biliary diseases, but the quality of all evidence was not high. Evidence from additional high-quality prospective studies are needed in the future.

**Supplementary Information:**

The online version contains supplementary material available at 10.1186/s12986-022-00677-1.

## Introduction

Gallbladder cancer and cholangiocarcinoma are major malignancies of the biliary tract. Additionally, according to anatomical position, bile duct cancer can be further divided into intrahepatic cholangiocarcinoma (iCCA) and extrahepatic cholangiocarcinoma (eCCA). Biliary cancer is one of the very lethal malignancies arising from the gallbladder or biliary duct epithelium, representing approximately 3–5% of all cancers of the gastrointestinal system [1]. Despite the low incidence of biliary cancers, cholangiocarcinoma is the second most common primary liver cancer after hepatocellular carcinoma (HCC), accounting for approximately 10% of primary liver cancers [1]. Although the treatment of biliary cancer has improved in recent years, long-term survival still needs to be improved, with a dismal 5-year survival rate of about 5% [2]. Biliary cancer can be related to chronic biliary tract or gallbladder inflammation owing to gallstone, choledocholithiasis, or primary sclerosing cholangitis, but the exact etiology remains poorly understood [3]. Clearly, for the general public, these risk factors do not provide appropriate recommendations for biliary tumor prevention.

Diet and nutrition, as modifiable risk factors, play an important role in the prevention of cardiovascular and cerebrovascular diseases [4], metabolic diseases, cancer etc. [5, 6]. For example, Guevara Cruz et al. [7] found that optimizing the diet pattern can reduce serum triglyceride and glucose tolerance of patients with metabolic syndrome. The randomized controlled trial conducted by Prentice et al. [8]. Confirmed that the low-fat diet pattern and can reduce the incidence rate of ovarian cancer in postmenopausal women. This also reveals that dietary factors may be related to the occurrence of malignant tumors. In addition, different dietary and nutritional factors have been proved to play a role in promoting or inhibiting cancer in malignant tumors of the biliary system [9]. For example, Larsson et al. [10] found that modified diet approach to stop hypertension (MDASH) diet and a modified Mediterranean (MMED) diet play a positive role in reducing the risk of extrahepatic BTC. A cohort study in Japan reported that fruit and vegetables intake tended to be associated with a reduced risk of eCCA [11].

Although there have been a number of meta-analyses summarizing evidence of the association between diet and nutrition-related factors (eg, daily foods, coffee, and alcohol) and the risk of biliary tract cancer, some evidence for the same factor varies considerably [12, 13]. Meanwhile, to date, there have been few comprehensive studies on the strength and quality of the evidence. Umbrella reviews provide a structured and critical summary of existing evidence, and enable the grading of evidence by specific criteria including sample size, strength and precision of the association, and assessment of the presence of biases. Hence, in order to better evaluate the existing evidence on the relationship between diet and nutrition-related indicators and biliary cancer risk, we conducted an umbrella review of the latest evidence from existing systematic reviews and meta-analyses.

In addition, cholecystolithiasis and other benign gallbladder diseases, as the most common disease of the biliary system (up to 20% of adults develop gallstones at some point in their lives [14]), is closely related to the occurrence of biliary tract tumors. As we all know, diet related factors are also closely related to the occurrence of gallstones. Therefore, we also included cholecystolithiasis/gallbladder diseases in this umbrella review to more comprehensively summarize the relationship between diet related factors and biliary diseases.

Previous studies have found that there are extensive links between drinking and common biliary diseases, and there are also some disputes. Therefore, we will elaborate drinking and biliary diseases as a separate topic.

## Methods

### Study design

Umbrella review is a summary of existing systematic review and meta-analysis, which aims to summarize the evidence from multiple studies around a research topic [15, 16]. We conducted this umbrella review to assess the relationship between diet and nutritional indicators and the risk of biliary tract diseases, such as gallbladder cancer, bile duct cancer, cholecystolithiasis or gallbladder diseases.

The protocol of this umbrella review was registered on PROSPERO (CRD42021293908).

### Literature search strategy

Two of the authors (Yaoqun Wang and Ningyuan Wen) independently conducted a comprehensive literature search using PubMed, Web of Science, and the Cochrane Database of Systematic Reviews. We searched studies published from database inception to 22 November 2021 to identify systematic reviews and meta analyses of retrospective or prospective studies.

The search algorithm used the following terms/keywords:

- Gallbladder cancer: (diet OR dietary OR food OR nutrition OR nutritional factors) AND (gallbladder cancer OR gallbladder carcinoma OR gallbladder neoplasms OR gallbladder tumor OR gallbladder neoplasm OR gallbladder mass OR gallbladder masses) AND (meta-analysis OR systematic review OR systematic overview).
- Bile duct cancer: (diet OR dietary OR food OR nutrition OR nutritional factors) AND (biliary Cancer OR biliary tumor OR biliary neoplasms OR biliary neoplasm OR biliary mass OR biliary masses OR cholangiocarcinoma OR bile duct cancer OR bile duct tumor OR bile duct neoplasms OR bile duct neoplasm OR bile duct mass OR bile duct masses) AND (meta-analysis OR systematic review OR systematic overview).
- Gallstone: (diet OR dietary OR food OR nutrition OR nutritional factors) AND (cholecystolithiasis OR gallstone OR gallbladder stone) AND (meta-analysis OR systematic review OR systematic overview).

Furthermore, manual searches on the reference lists of the identified publications, references of other nutrition related umbrella reviews and research registration platform were also conducted to identify additional studies relevant to our umbrella review. Disagreements were resolved by discussion between the two authors. Detailed search strategies and manual searches results can be found in Additional file 1: Table S1.

### Selection and exclusion criteria

The topic of our study is the association between diet and nutrition-related factors and disease, which is not applicable to randomized controlled studies. Hence, our study mainly included systematic reviews and meta-analyses based on cohort or case–control studies.

The inclusion criteria were as follows: (1) Meta analyses and systematic reviews of retrospective or prospective studies adhering to PRISMA guidelines; (2) Evaluated the association of diet and nutrition related factors and risk of biliary tract disease. Eligible dietary factors included daily foods, beverages (including alcohol) etc. Eligible nutrition related indicators included BMI, glycemic index (including diabetes), blood lipids etc.; (3) Summarized and reported Odds Ratios(OR), Relative Rates(RR) or Hazard Ratios (HR) and corresponding 95% confidence interval (CI) from studies; (4) No participants and language restriction were used in the selection of eligible studies; (5) Whenever there were multiple health outcomes, we only extract the data of the diseases concerning biliary system; (6) If there are multiple meta-analysis and/or systematic review on the same topic, the most recent study with the largest number of studies and effect size was included.

The exclusion criteria were: (1) Animal studies; (2) Narrative reviews, original studies, conference proceedings and letters to editors; (3) Systematic reviews or meta-analyses targeting other non-biliary diseases; (4) Studies in which diet, nutritional factors, or nutritional indicators were not the exposure of interest; (5) Studies that did not provide study specific data: Odds Ratios(OR), Relative Rates(RR) or Hazard Ratios (HR) and corresponding 95% confidence interval (CI).

### Data extraction

Two authors (Yaoqun Wang and Ningyuan Wen) extracted data separately.

Any disagreement in the extracted data was re-evaluated by a third author (Jiong Lu). For each eligible systematic review and meta-analysis, we first extracted the following general characteristics: (1) the first author; (2) the publication year; (3) original article retrieval time; (4) journal; (5) dietary factor or nutrition related indicators in the study; (6) number of studies included;(7) outcomes of interest investigated (disease type), country or region of original studies and the number of corresponding studies; (8) study design(cohort, case–control, cross-sectional, Nested case–control); (9) number of cases/total participants; (10) quality assessment of each eligible systematic review or meta-analysis.

Furthermore, the main findings of each study were also abstracted: (1) the type of effect model; (2) meta-analysis metric; (3) estimated summary effect (OR: Odds Ratios, RR: Relative Rates or HR: Hazard Ratios), 95% confidence intervals (CIs) and p-value of test for estimated summary effect; (4) heterogeneity (*I* ^2^) and p-value; (5) publication bias by Egger's test and small study effect; (6) subgroup analyses; (7) type of comparison (e.g. high vs. low analysis or dose–response analysis) was abstracted when possible.

### Quality evaluation

AMSTAR2 is a practical tool for evaluating the quality of systematic reviews and meta analyses. The revised AMSTAR2 [17] consists of 16 items, which covers the whole process of systematic reviews and meta-analyses, including topic selection, design, registration, data extraction, statistical analysis and discussion. The details of AMSTAR2 scale are shown in Table 5. Among these 16 items, items 2, 4, 7, 9, 11, 13 and 15 are critical items. The detailed grading criteria of AMSTAR2 scale for systematic review and meta-analysis are as follows [17]:

- High: No or one non-critical weakness: the systematic review provides an accurate and comprehensive summary of the results of the available studies that address the question of interest [17].
- Moderate: More than one non-critical weakness*: the systematic review has more than one weakness but no critical flaws. It may provide an accurate summary of the results of the available studies that were included in the review [17].
- Low: One critical flaw with or without non-critical weaknesses: the review has a critical flaw and may not provide an accurate and comprehensive summary of the available studies that address the question of interest [17].
- Critically low: More than one critical flaw with or without non-critical weaknesses: the review has more than one critical flaw and should not be relied on to provide an accurate and comprehensive summary of the available studies [17].

When evaluating the quality of evidence,the GRADE [18, 19] was adopted to rate the strength of evidence for each outcome in each meta-analysis. According to the GRADE classification, evidence from randomized controlled trials was defined as high quality without degradation, while evidence from observational studies is automatically reduced by two levels at the beginning, defined as low-quality evidence. Next, evidence was comprehensively evaluated according to five factors that may lead to the reduction of evidence quality (risk of bias, Indirectness, inconsistency, imprecision and publication bias) and three factors that may upgrade evidence quality (large effect, dose–response gradient and plausible confounding). Finally, evidence was divided into four levels according to its quality (high, moderate, low and very low).

### Statistical analysis

For each meta-analysis included in our study, we abstracted exposure, outcome and the estimated summary effect (OR: Odds Ratios, RR: Relative Rates or HR: Hazard Ratios) with its corresponding 95% CI and *p*-value. Cochran's Q test and the* I*^2^ metric were used to assess the heterogeneity between different studies. The selection of random or fixed effect models was adopted from the original models in the selected meta-analyses. We did not conduct a secondary analysis. Egger’s test [20] was used to calculate publication bias or small study effect, and when *p*-value < 0.1, we considered occurrence of publication bias. For other statistical tests, the significance threshold was still set as *p* < 0.05. The dose–response analyses were abstracted from the articles when possible.

## Results

### Characteristics of the included meta-analyses

**Fig. 1:**
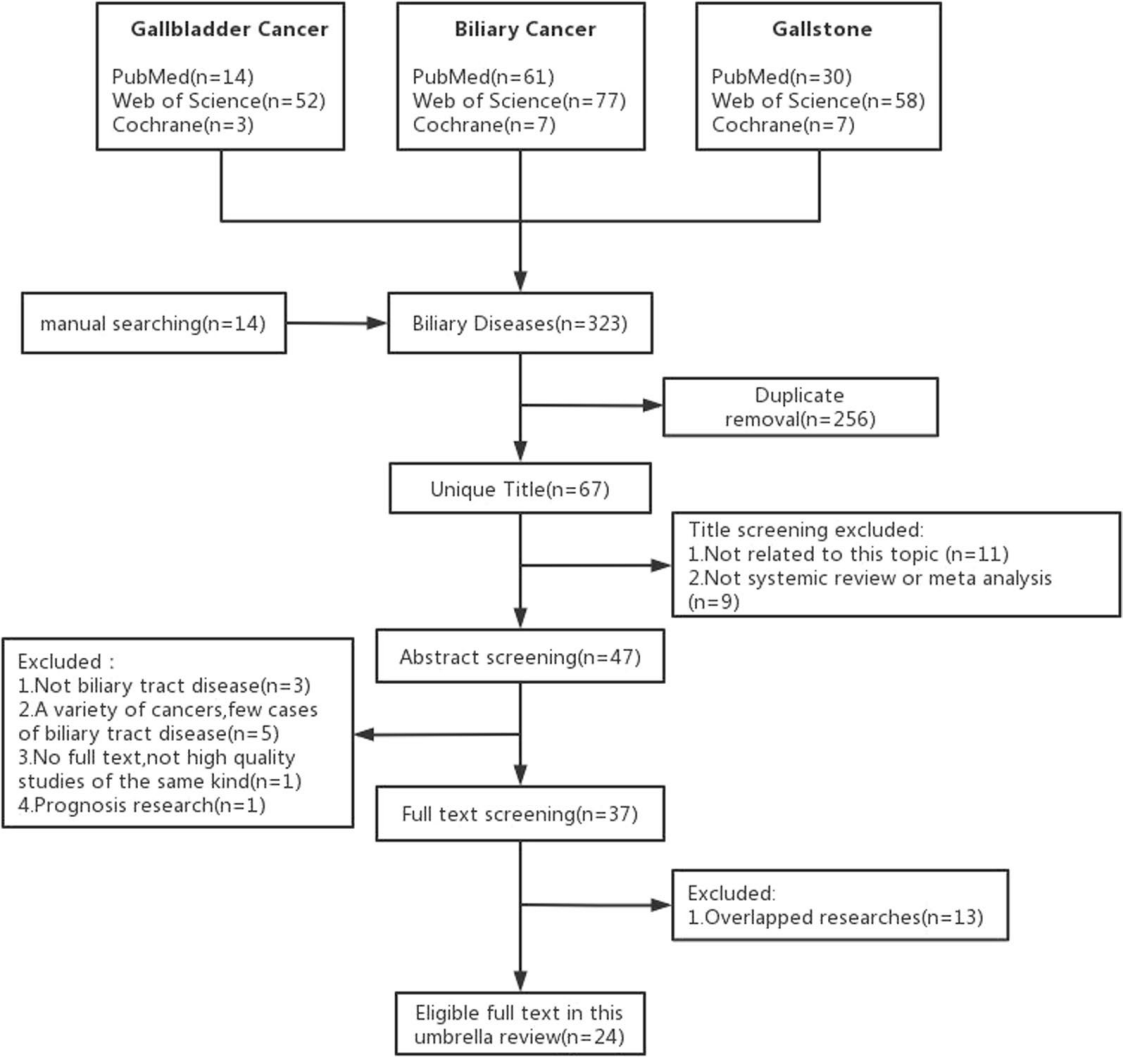
Literature screening process of this study

The process of literature screening is shown in Fig. 1. Two authors independently and systematically retrieved 323 articles respectively. Overall, 24 articles with 83 unique outcomes were included by eligibility criteria. A list of all the excluded articles and the reason for exclusion was provided in Additional file 2: Table S2.

Table 1 shows the characteristics of these 24 studies. Among all included articles, nine did not conduct methodological quality assessment for original studies [21–28], the remaining 15 articles used the Newcastle Ottawa Quality Assessment scale(NOS) for assessment. The included studies covered 43 significant associations between 83 diet, nutrition related indicators and biliary diseases risk. All systematic reviews and meta-analyses were published between 2008 and 2021. Among these articles, four included only case–control studies [1, 27, 29–31], six included only cohort studies [25, 26, 32–35], and the remaining thirteen included different types of original studies, encompassing case–control studies, cohort studies, nested case–control studies and cross-sectional studies. The number of original studies included in these articles ranged from 2 to 26. Except that only one article could not obtain the exact number of participants and cases [24], the number of participants in other studies were at least 435, at most 10,786,685, and the number of cases were at least 105, at most 61,071.

**Table 1:** The general characteristics of the 24 systematic reviews and meta-analyses

First author, year	Original article retrieval time	Journal	Dietary factor or nutrition related indicators	No. of studies included in this review related to our topic	Disease type; continent/region/country; no. of studies	Type of studies	Study design(number)	Sample size	Quality assessment
Bagnardi [21]	Up to September 2012	British Journal of Cancer	Alcohol	8 studies	GBC; North America(3), Asia(5)	Meta-analysis	Cohort(4) Case–control(4)	880 GBC cases	NA
Clements [1]	NA	Journal of Hepatology	Alcohol	15 studies	iCCA; China(5);America(5);South Korea(2); Denmark(1); Italy(1); Japan(1)	Systematic review and meta-analysis	Case–control(15)	13,986 cases and 780,565 controls	NOS
			Alcohol	11 studies	eCCA: China(6);South Korea(1)		Case–control(11)	8,293 cases and 452,450 controls	
Godos [13]	Up to March 2017	Nutrients	Coffeee	5 publications on 17 studies	BTC; America(11);Japan(3); Sweden(3)	Meta-analysis of prospective cohort studies	Cohort(15) Case–control(2)	726 cases among 1,375,626 participants	NOS
Chen [29]	Up to June 2017	Chinese Medical Journal	High Spicy Food	3 publications on 6 studies	GBC; Hungary(2),Chile(2),India(2)	Meta-analysis of case–control studies	Case–control(6)	219 cases and 245 controls	NOS
Xiong [36]	NA	Oncotarget	Tea	8 studies	BTC; West(4);East(4)	Systematic review and meta-analysis	Cohort(3) Case–control(5)	7968 BTC cases	NOS
ZHU [22]	NA	Molecular and Clinical Oncology	Tea	6 studies	GBC; China(2), America(1), Italy(1), Japan(1),Poland(1)	Meta-analysis	Cohort(2) Case–control(4)	753 cases among 115,349 participants	NA
Huai [37]	Up to 31 May 2020	Nutrition and Cancer-an International Journal	Vegetable	10 studies	BTC; Thailand(4),Japan(2),Italy(1),Netherland(1), Hungary(1), India(1)	Meta-analysis	Case–control(8) Cohort(1) Nested case–control(1)	2620 cases among 90,829 participants	NOS
			Fruit	13 studies	BTC; Thailand(5),Japan(2), India(2),Italy(1),Chili(1), Nepal(1), Hungary(1)		Case–control(11) Cohort(1) Nested case–control(1)	2926 cases among 90,866 participants	
Kamsa-ard [30]	Up to 4 March 2016	Asian Pacific Journal of Cancer Prevention	Raw Fish	3 studies	CCA; Thailand(3)	Systematic review and meta-analysis	Case–control(3)	1920 participants	NOS
			Fermented Fish	2 studies	CCA; Thailand(2)		Case–control(2)	435 participants	
			Glutinous Rice	3 studies	CCA; Thailand(3)		Case–control(3)	842 participants	
			Meat	2 studies	CCA; Thailand(2)		Case–control(2)	616 participants	
			Betel nut	3 studies	CCA; Thailand(3)		Case–control(3)	709 participants	
Steele [23]	Up to 8 February 2015	Infectious Diseases of Poverty	Fermented Meats	3 studies	CCA; Thailand(3)	Systematic review and meta-analysis	Case–control(2) Nested case–control(1)	471 cases and 690 controls	NA
			High Nitrate Foods	5 studies	CCA; Thailand(5)		Case–control(3) Nested case–control(2)	682 cases and 901 controls	
			Rice	2 studies	CCA; Thailand(2)		Case–control(2)	232 cases and 232 controls	
Daniel [26]	NA	Scandinavian Journal of Gastroenterology	Triglycerides	2 studies	Gallstone; Denmark(1),Sweden(1)	a cohort study and a systematic review with meta analysis	Cohort(2)	298 cases among 3038 participants	NA
			HDL cholesterol						
			Non-HDL cholesterol						
Byung [38]	Up to 01 March 2018	Gut and Liver	Alcohol	24 studies	Gallstone; America and Europe(19), Asia(4),Australia(1)	Meta-analysis of case–control and cohort Studies	Cohort(9) Case–control(15)	22,401 cases among 76,185 participants	NOS
Zhang [39]	Up to June 2015	Alimentary Pharmacology & Therapeutics	Coffee	6 publications on 8 studies	Gallstone; America(2),Italy(2),Sweden(1),Britain(1)	Systematic review and meta-analysis	Cohort(7) Case–control(1)	11,477 cases among 227,749 participants	NOS
Zhang [40]	Up to March 2018	Medicine	Vegetable	14 studies	Gallstone; America(5),Italy(1),Iran(1), French(1),Sweden(1), Britain(1),Korea(1),Indian(1),Germany(1), Argentina(1)	Systematic review and meta-analysis	Cohort(9) Case–control(4) Cross-sectional(1)	33,983 cases among 1,533,752 participants	NOS
			Fruit	5 studies	Gallstone; America(2),French(1),Sweden(1),Korea(1)		Cohort(4) Case–control(1)	20,599 cases among 1,223,147 participants	
Ying Li [31]	Up to February 2010	PLOS ONE	Alcohol	2 studies	GBC; China(2)	Systematic review and meta-analysis	Case–control(2)	467 cases and 1315 controls	NOS
				2 studies	VPC; China(2)		Case–control(2)	105 cases and 1331 controls	
				2 studies	eCCA; China(2)		Case–control(2)	228 cases and 753 controls	
Emma E. McGee [27]	NA	Jnci-Journal of the National Cancer Institute	Alcohol	Total 26 studies	GBC; NA	A Pooling Project and meta-analysis	Cohort(26)	1104 cases among 230,0628 participants	NA
				Total 26 studies	iCCA; NA			613 cases among 230,0628 participants	
				Total 26 studies	eCCA; NA			928 cases among 230,0628 participants	
				Total 26 studies	VPC; NA			521 cases among 230,0628 participants	
Xiao-Hua Ye [35]	Up to 31 May 2013	World Journal of Gastroenterology	Alcohol	7 studies	eCCA; America(3); China(3); Turkey(1)	Meta-analysis	Case–control(6) Cohort(1)	783 cases among 1770 participants	NOS
Jiantao Wang [28]	Up to May 2016	European Journal of Gastroenterology &Hepatology	Alcohol	18 studies	Gallstone; Europe(11); America(3); Asia(2); Oceania(2)	Meta-analysis	Case–control(10) Cohort(8)	29,680 cases among 415,747 participants	NA
Gu [41]	Up to 31 August 2014	Diabetes-Metabolism Research and Reviews	T2DM	20 studies	GBC; East Asia(6),America(6), Europe(6),India (n = 1), Israel (n = 1),	Systematic review and meta-analysis of observational studies	Cohort(12) Case–control(8)	4106 cases among 4,223,350 participants	NOS
Li [42]	Up to August 2015	Obesity	Overweight	17 studies	GBC; Europe(7),Asia(7),Americas(3)	Meta-analysis of observational studies	Cohort(9) Case–control(8)	6285 cases among 6,183,691 participants	NOS
			Obesity	22 studies	GBC; Europe(10),Asia(8),Americas(4)		Cohort(13) Case–control(9)	6761 cases among 10,786,685 participants	
			Overweight	8 studies	eCCA; Europe(4),Asia(3),Americas(1),		Cohort(4) Case–control(4)	1938 cases among 1,614,375 participants	
			Obesity	16 studies	eCCA; Europe(6),Americas(6),Asia(4)		Cohort(7) Case–control(9)	5819 cases among 6,479,962 participants	
Aune [33]	Up to 9 January 2015	Journal of Diabetes and its Complications	DM	10 studies	GBD; America(6),China(2),Italy(1),Britain(1)	Systematic review and meta-analysis of prospective studies	Cohort(10)	223,651 cases among 7,365,198 participants	NOS
Barclay [25]	Up to March 2007	American Journal of Clinical Nutrition	Glycemic index rate	2 studies	Gallstone:America(2)	Meta analysis	Cohort(2)	114,933 participants	NA
			Glycemic load rate						
Dagfinn Aune [34]	Up to 9 January 2015	European Journal of Epidemiology	BMI	17 studies	GBD; America(9),Europe(7),China(1)	Meta analysis	Cohort(17)	55,670 cases among 1,921,103 participants	NOS
			Waist Circumference	5 studies	GBD; America(4),Europe(1)		Cohort(5)	15,523 cases among 284,095 participants	
			Waist-to-Hip Ratio	4 studies	GBD; America(4)		Cohort(4)	14,458 cases among 230,166 participants	
Petrick [24]	Up to 5 September 2017	American Journal of Gastroenterology	Obesity	4 studies	iCCA; America(3),Europe(1)	Pooling Project and meta-analysis	Cohort(1) Nested case–control(3)	NA	NA
			DM	6 studies	iCCA; America(3),Europe(2),Asia(1)		Cohort(2) Nested case–control(4)	NA	

Tables 2, 3 and 4 respectively show the relationship between dietary factors, nutrition related indicators, alcohol and biliary diseases. Among the included articles, 4 focused on gallbladder cancer, 8 on cholangiocarcinoma, 9 on cholecystolithiasis/gallbladder diseases, and 3 on both gallbladder cancer and cholangiocarcinoma. Seventeen of twenty-four studies focused on fourteen food items, including alcohol, coffeee, high spicy food, tea, vegetable, fruit, raw fish, fermented fish, glutinous rice, meat, betel nut, fermented meats, high nitrate foods and rice. Seven studies focused on 11 nutrition related indicators, including overweight, obesity, diabetes mellitus, glycemic index rate, glycemic load rate, every 5 unit increment of BMI, every 10 cm increment of waist circumference, every 0.1 unit increment in waist-to-hip ratio, triglycerides, HDL cholesterol and Non-HDL cholesterol.

**Table 2:** The relationship between dietary factors and biliary diseases

First author, Year	No. of included studies	Type of study	Dietary factor (Subgroup or Dose response)	Effects model	MA metric	Estimates	95%CI	Test for overall effect (p-value)	*I2*% (p-value)	Egger test (p-value)	Publication bias and small-study effect
*Gallbladder cancer*											
Chen [29]	6	Case–control	All spicy food	random	OR	1.78	(0.83–3.83)	NA	75 (0.001)	0.714	No publication bias
Chen [29]	6	Case–control	Chili pepper	random	OR	1.78	(0.83–3.83)	NA	75 (0.001)	0.714	No publication bias
ZHU [22]	6	Case–control(4);Cohort(2)	Tea	random	OR	0.67	(0.40–1.12)	0.13	82 (< 0.0001)	Only funnel plot (N)	No publication bias
ZHU [22]	4	Case–control(3);Cohort(1)	Tea (highest vs. lowest/none)	random	OR	0.57	(0.25–1.29)	0.18	82 (0.001)	Only funnel plot (N)	No publication bias
ZHU [22]	4	Case–control(3);Cohort(1)	Tea (moderate vs. low/none)	random	OR	0.62	(0.33–1.14)	0.12	77 (0.004)	Only funnel plot (N)	No publication bias
*Biliary tract cancer*											
Godos [13]	8	Total Cohort(5);Case–control(3)	Coffee	random	OR	0.83	(0.64–1.08)	NA	0 (0.58)	Only funnel plot (N)	No publication bias
Godos [13]#	5	Cohort(5)	Coffee	random	OR	0.74	(0.34–1.63)	NA	0 (0.82)	Only funnel plot (N)	No publication bias
Godos [13]#	3	Case–control(3)	Coffee	random	OR	0.84	(0.61–1.15)	NA	22 (0.27)	Only funnel plot (N)	No publication bias
Xiong [36]	8	Total Case–control(5);Cohort(3)	Tea	random	RR	0.66	(0.48–0.85)	NA	81.1 (0.000)	> 0.05	No publication bias
Xiong [36]#	3	Cohort	Tea	random	RR	0.62	(0.44–0.80)	NA	55.8 (0.009)	> 0.05	No publication bias
Xiong [36]#	5	Case–control	Tea	random	RR	0.84	(0.77–0.90)	NA	60 (0.001)	> 0.05	No publication bias
Xiong [36]	8	Total Case–control(5);Cohort(3)	Tea (every 1cup/day increment)	–	RR	0.96	(0.93–0.98)	0.001	NA	> 0.05	No publication bias
Huai [37]	10	Total Case–control(8);Cohort(1); Nested case–control(1)	Vegetable	random	RR	0.48	(0.22–0.74)	NA	86.8 (0.000)	0.84	No publication bias
Huai [37]#	8	Case–control	Vegetable	random	RR	0.45	(0.14–0.75)	NA	88 (< 0.001)	0.84	No publication bias
Huai [37]#	1	Cohort	Vegetable	–	RR	0.96	(0.37–1.55)	NA	–	0.84	No publication bias
Huai [37]#	1	Nested case–control	Vegetable	–	RR	0.40	(0.23–0.76)	NA	–	0.84	No publication bias
Huai [37]	8	Case–control(6);Cohort(1); Nested case–control(1)	Vegetable (every 100 g/day increment)	–	RR	0.31	(0.20–0.47)	< 0.001	NA	0.84	No publication bias
Huai [37]	13	Total Case–control(11);Cohort(1); Nested case–control(1)	Fruit	random	RR	0.47	(0.32–0.61)	NA	63.3 (0.001)	0.64	No publication bias
Huai [37]#	11	Case–control	Fruit	random	RR	0.41	(0.26–0.56)	NA	61.6 (0.004)	0.64	No publication bias
Huai [37]#	1	Cohort	Fruit	–	RR	0.87	(0.47–1.27)	NA	–	0.64	No publication bias
Huai [37]#	1	Nested case–control	Fruit	–	RR	0.60	(0.33–0.98)	NA	–	0.64	No publication bias
Huai [37]	8	Case–control(6);Cohort(1); Nested case–control(1)	Fruit (every 100 g/day increment)	–	RR	0.89	(0.66–1.18)	0.35	NA	0.64	No publication bias
Kamsa-ard [30]	3	Case–control	Raw Fish	fixed	OR	2.54	(1.94–3.35)	< 0.00001	0 (0.80)	NA	NA
Kamsa-ard [30]	2	Case–control	Fermented Fish	fixed	OR	1.61	(0.76–3.41)	0.21	45 (0.18)	NA	NA
Kamsa-ard [30]	3	Case–control	Glutinous Rice	fixed	OR	1.30	(0.85–2.01)	0.23	62 (0.07)	NA	NA
Kamsa-ard [30]	2	Case–control	Meat	random	OR	1.03	(0.57–1.85)	0.92	59 (0.06)	NA	Na
Kamsa-ard [30]	3	Case–control	Betel nut	fixed	OR	1.45	(0.69–3.02)	0.33	60 (0.06)	NA	NA
Steele [23]	3	Total Case–control(2) Nested case–control(1)	Fermented Meats	random	OR	1.81	(0.96–3.39)	0.066	17 (0.28)	NA	NA
Steele [23]	5	Total Case–control(3) Nested case–control(2)	High Nitrate Foods	random	OR	1.41	(1.05–1.91)	0.024	46 (0.01)	NA	NA
Steele [23]	2	Case–control	Rice	random	OR	0.88	(0.48–1.63)	0.688	34 (0.22)	NA	NA
*Cholecystolithiasis/gallbladder diease*											
Zhang [39]	7	Cohort	Coffee	random	RR	0.83	(0.76–0.89)	NA	35.9 (0.154)	0.39	No publication bias
Zhang [39]	4	Cohort	Coffee (every 1Cup/Day increment)	–	RR	0.95	(0.91–1.00)	0.049	54.4 (0.019)	0.39	No publication bias
Zhang [40]	14	Total Case–control(4);Cohort(9);Cross sectional(1)	Vegetables	random	RR	0.83	(0.74–0.94)	NA	82.5 (0.000)	0.682	No publication bias
Zhang [40]#	9	Cohort	Vegetables	random	RR	0.92	(0.82–1.02)	NA	80.2 (0.001)	0.682	No publication bias
Zhang [40]#	4	Case–control	Vegetables	random	RR	0.39	(0.24–0.62)	NA	59.8 (0.058)	0.682	No publication bias
Zhang [40]#	1	Cross sectional	Vegetables	random	RR	0.92	(0.80–1.07)	NA	–	0.682	No publication bias
Zhang [40]	6	Cohort	Vegetables(every 200 g/Day increment)	–	RR	0.96	(0.93–0.98)	0.001	NA	0.682	No publication bias
Zhang [40]	5	Cohort	Fruits	random	RR	0.88	(0.83–0.92)	NA	0 (0.456)	0.735	No publication bias
Zhang [40]	4	Cohort	Fruits (every 200 g/Day increment)	–	RR	0.97	(0.96–0.98)		NA	0.735	No publication bias

#: Subgroup analysis of the different study design types of the corresponding study

**Table 3:** The relationship between nutrition related indicators and biliary diseases

First author, year	No. of included studies	Type of study	Nutrition related indicators	Effects model	MA metric	Estimates	95%CI	Test for overall effect (*p*-value)	*I2%* (*p*-value)	Egger test (*p*-value)	Publication bias and small-study effect
*Gallbladder cancer*											
Gu [41]	20	Total Case–control(8); Cohort(12)	Type 2 DM	random	RR	1.56	(1.36–1.79)	NA	43.5 (0.01)	< 0.001	Exist publication bias
Gu [41]#	8	Case–control	Type 2 DM	random	RR	1.52	(1.09–2.11)	NA	38.8 (0.109)	< 0.001	Exist publication bias
Gu [41]#	12	Cohort	Type 2 DM	random	RR	1.57	(1.35–1.83)	NA	48.7 (0.013)	< 0.001	Exist publication bias
Li [42]	17	Total Case–control(8); Cohort(9)	Overweight	random	RR	1.17	(1.07–1.28)	NA	32.6 (0.03)	0.375	No publication bias
Li [42]#	8	Case–control	Overweight	random	RR	1.24	(1.07–1.44)	NA	0 (0.877)	0.375	No publication bias
Li [42]#	9	Cohort	Overweight	random	RR	1.15	(1.02–1.30)	NA	56.9 (0.004)	0.375	No publication bias
Li [42]	22	Total Case–control(9); Cohort(13)	Obesity	random	RR	1.62	(1.49–1.75)	NA	0 (0.524)	0.375	No publication bias
Li [42]#	9	Case–control	Obesity	random	RR	1.48	(1.26–1.74)	NA	0 (0.544)	0.375	No publication bias
Li [42]#	13	Cohort	Obesity	random	RR	1.67	(1.52–1.83)	NA	0 (0.492)	0.375	No publication bias
*Biliary tract cancer*											
Li [42]	8(eCCA)	Total Case–control(4); Cohort(4)	Overweight	random	RR	1.26	(1.14–1.39)	NA	0 (0.452)	0.478	No publication bias
Li [42]#	4(eCCA)	Case–control	Overweight	random	RR	1.11	(0.89–1.39)	NA	10.3 (0.350)	0.478	No publication bias
Li [42]#	4(eCCA)	Cohort	Overweight	random	RR	1.31	(1.16–1.47)	NA	0 (0.596)	0.478	No publication bias
Li [42]	16(eCCA)	Total Case–control(9); Cohort(7)	Obesity	random	RR	1.48	(1.21–1.81)	NA	68 (0.000)	0.478	No publication bias
Li [42]#	9(eCCA)	Case–control	Obesity	random	RR	1.27	(1.03–1.55)	NA	53.6 (0.011)	0.478	No publication bias
Li [42]#	7(eCCA)	Cohort	Obesity	random	RR	1.81	(1.29–2.53)	NA	62.7 (0.004)	0.478	No publication bias
Petrick [24]	4(iCCA)	Total Nested case–control(3); Cohort(1)	Obesity	random	RR	1.49	(1.32–1.70)	< 0.001	0 (0.70)	0.09	Exist publication bias
Petrick [24]#	3(iCCA)	Nested case–control	Obesity	random	RR	1.46	(1.27–1.69)	< 0.001	0 (0.60)	0.09	Exist publication bias
Petrick [24]#	1(iCCA)	Cohort	Obesity	random	RR	1.62	(1.23–2.12)	< 0.001	–	0.09	Exist publication bias
Petrick [24]	6(iCCA)	Total Nested case–control(4); Cohort(2)	DM	random	RR	1.53	(1.31–1.78)	< 0.001	67.3 (0.009)	0.9	No publication bias
Petrick [24]#	4(iCCA)	Nested case–control	DM	random	RR	1.59	(1.47–1.72)	< 0.001	0 (0.40)	0.9	No publication bias
Petrick [24]#	2(iCCA)	Cohort	DM	random	RR	1.45	(0.99–2.13)	0.06	81.1 (0.02)	0.9	No publication bias
*Cholecystolithiasis/gallbladder diease*											
Aune [33]	10	Cohort	DM	random	RR	1.56	(1.26–1.93)	NA	99.4 (< 0.0001)	0.70	No publication bias
Barclay [25]	2	Cohort	Glycemic index rate (highest vs. lowest)	fixed	RR	1.26	(1.13–1.40)	< 0.0001	NA	Only funnel plot (N)	No publication bias
Barclay [25]	2	Cohort	Glycemic load rate (highest vs. lowest)	fixed	RR	1.41	(1.25–1.60)	< 0.0001	NA	Only funnel plot (N)	No publication bias
Dagfinn Aune [34]	17	Cohort	Every 5 unit increment of BMI	random	RR	1.63	(1.49–1.78)	NA	98 (< 0.0001)	0.13	No publication bias
Dagfinn Aune [34]	5	Cohort	Every 10 cm increment of waist circumference	random	RR	1.46	(1.24–1.72)	NA	98 (< 0.0001)	NA	NA
Dagfinn Aune [34]	4	Cohort	Every 0.1 unit increment in waist-to-hip ratio	random	RR	1.44	(1.26–1.64)	NA	92 (< 0.0001)	NA	NA
Daniel [26]	2	Cohort	Triglycerides	fixed	OR	1.10	(0.99–1.22)	NA	0 (NA)	NA	NA
Daniel [26]	2	Cohort	HDL cholesterol	fixed	OR	0.87	(0.62–1.23)	NA	0 (NA)	NA	NA
Daniel [26]	2	Cohort	Non-HDL cholesterol	fixed	OR	1.19	(1.07–1.32)	NA	81 (NA)	NA	NA

#: Subgroup analysis of the different study design types of the corresponding study

**Table 4:** The relationship between alcohol consumption and biliary diseases

First author, Year	No. of included studies	Type of study	Dietary factor (subgroup or dose response)	Effects model	MA metric	Estimates	95%CI	Test for overall effect (*p*-value)	*I2*% (*p*-value)	Egger test (*p*-value)	Publication bias and small-study effect
*Gallbladder cancer*											
Bagnardi [16]	8	Case–control(4); Cohort(4)	Alcohol (Light vs. none)^c^	Random	RR	1.23	(0.84 − 1.83)	NA	18 (NA)	NA	NA
Bagnardi [16]	8	Case–control(4); Cohort(4)	Alcohol (Light vs. none)^c^	Random	RR	0.88	(0.68 − 1.13)	NA	10 (NA)	NA	NA
Bagnardi [16]	8	Case–control(4); Cohort(4)	Alcohol (Light vs. none)^c^	Random	RR	2.64	(1.62 − 4.30)	NA	0 (NA)	NA	NA
Ying Li [50]	2	Case–control	Alcohol (drinker vs. non-drinker)	Fixed	OR	0.7	99%CI(0.49–1.00)	0.009	16 (0.27)	NA	NA
Emma E. McGee [35]	Total 26 studies	Cohort	Alcohol (> 0–0.5 vs. 0 drink/d)^a^	Random	HR	1.07	(0.91–1.26)	NA	0 (NA)	NA	NA
Emma E. McGee [35]	Total 26 studies	Cohort	Alcohol (> 0.5–1 vs. 0 drink/d)^a^	Random	HR	1.1	(0.87–1.39)	NA	0 (NA)	NA	NA
Emma E. McGee [35]	Total 26 studies	Cohort	Alcohol (1– < 3 vs. 0 drink/d)^a^	Random	HR	0.94	(0.74–1.21)	NA	0 (NA)	NA	NA
Emma E. McGee [35]	Total 26 studies	Cohort	Alcohol (3– < 5, vs. 0 drink/d)^a^	Random	HR	1.16	(0.69–1.94)	NA	0 (0.57)	NA	NA
Emma E. McGee [35]	Total 26 studies	Cohort	Alcohol (> 5 vs. 0 drink/d)^a^	Random	HR	2.39	(0.63–9.12)	NA	64.9 (0.02)	NA	NA
Emma E. McGee [35]	Total 26 studies	Cohort	Alcohol (every 1drink/d increment)^a^	–	HR	0.98	(0.92–1.05)	0.31	12 (NA)	NA	NA
*Biliary tract cancer*											
Ying Li [50]	2(eCCA)	Case–control	Alcohol (drinker vs. non-drinker)	Fixed	OR	1.14	99%CI(0.75–1.75)	0.41	0 (0.81)	NA	NA
Ying Li [50]	2(VPC)	Case–control	Alcohol (drinker vs. non-drinker)	Random	OR	0.68	99%CI(0.20–2.37)	0.43	77 (0.04)	NA	NA
Emma E. McGee [35]	Total 26 studies(iCCA)	Cohort	Alcohol (> 0–0.5 vs. 0 drink/d)^a^	Random	HR	0.79	(0.62–1.00)	NA	0 (NA)	NA	NA
Emma E. McGee [35]	Total 26 studies(iCCA)	Cohort	Alcohol (> 0.5–1 vs. 0 drink/d)^a^	Random	HR	0.91	(0.65–1.26)	NA	0 (NA)	NA	NA
Emma E. McGee [35]	Total 26 studies(iCCA)	Cohort	Alcohol (1– < 3 vs. 0 drink/d)^a^	Random	HR	0.98	(0.73–1.31)	NA	0 (NA)	NA	NA
Emma E. McGee [35]	Total 26 studies(iCCA)	Cohort	Alcohol (3– < 5, vs. 0 drink/d)^a^	Random	HR	1.25	(0.77–2.02)	NA	8.5 (0.37)	NA	NA
Emma E. McGee [35]	Total 26 studies(iCCA)	Cohort	Alcohol (> 5 vs. 0 drink/d)^a^	Random	HR	2.35	(1.46–3.78)	NA	0 (0.52)	NA	NA
Emma E. McGee [35]	Total 26 studies(iCCA)	Cohort	Alcohol (every 1drink/d increment)^a^	–	HR	1.03	(1.01–1.06)	0.04	0 (NA)	NA	NA
Emma E. McGee [35]	Total 26 studies(eCCA)	Cohort	Alcohol (> 0–0.5 vs. 0 drink/d)^a^	Random	HR	0.87	(0.68–1.12)	NA	30.8 (NA)	NA	NA
Emma E. McGee [35]	Total 26 studies(eCCA)	Cohort	Alcohol (> 0.5–1 vs. 0 drink/d)^a^	Random	HR	1.14	(0.82–1.58)	NA	28.4 (NA)	NA	NA
Emma E. McGee [35]	Total 26 studies(eCCA)	Cohort	Alcohol (1– < 3 vs. 0 drink/d)^a^	Random	HR	1.08	(0.74–1.58)	NA	48.3 (NA)	NA	NA
Emma E. McGee [35]	Total 26 studies(eCCA)	Cohort	Alcohol (3– < 5, vs. 0 drink/d)^a^	Random	HR	1.82	(0.98–3.39)	NA	57.2 (0.01)	NA	NA
Emma E. McGee [35]	Total 26 studies(eCCA)	Cohort	Alcohol (> 5 vs. 0 drink/d)^a^	Random	HR	1.02	(0.64–1.62)	NA	0 (0.84)	NA	NA
Emma E. McGee [35]	Total 26 studies(eCCA)	Cohort	Alcohol (every 1drink/d increment)^a^	–	HR	1.03	(0.98–1.08)	0.84	25.3 (NA)	NA	NA
Emma E. McGee [35]	Total 26 studies(VPC)	Cohort	Alcohol (> 0–0.5 vs. 0 drink/d)^a^	Random	HR	1.08	(0.80–1.45)	NA	13.7 (NA)	NA	NA
Emma E. McGee [35]	Total 26 studies(VPC)	Cohort	Alcohol (> 0.5–1 vs. 0 drink/d)^a^	Random	HR	0.99	(0.69–1.41)	NA	0 (NA)	NA	NA
Emma E. McGee [35]	Total 26 studies(VPC)	Cohort	Alcohol (1– < 3 vs. 0 drink/d)^a^	Random	HR	1.33	(0.99–1.80)	NA	0 (NA)	NA	NA
Emma E. McGee [35]	Total 26 studies(VPC)	Cohort	Alcohol (3– < 5, vs. 0 drink/d)^a^	Random	HR	1.16	(0.66–2.01)	NA	0 (0.93)	NA	NA
Emma E. McGee [35]	Total 26 studies(VPC)	Cohort	Alcohol (> 5 vs. 0 drink/d)^a^	Random	HR	1.59	(0.85–2.98)	NA	0 (0.73)	NA	NA
Emma E. McGee [35]	Total 26 studies(VPC)	Cohort	Alcohol (every 1drink/d increment)^a^	–	HR	1.00	(0.95–1.04)	0.35	0 (NA)	NA	NA
Xiao-Hua Ye [51]	7(eCCA)	Total case–control(6);Cohort(1)	Alcohol (drinker vs. non-drinker)	Random	RR	1.09	(0.87–1.37)	NA	0 (0.575)	0.296	No publication bias
Xiao-Hua Ye [51]#	6(eCCA)	Case–control(6)	Alcohol (drinker vs. non-drinker)	Random	RR	1.10	(0.86–1.41)	NA	0 (0.447)	0.296	No publication bias
Xiao-Hua Ye [51]#	1(eCCA)	Cohort(1)	Alcohol (drinker vs. non-drinker)	Random	RR	1.06	(0.60–1.87)	NA	–	0.296	No publication bias
Clements [1]	15(iCCA)	Case–control	Alcohol (drinker vs. non-drinker)	Random	OR	3.15	(2.24–4.41)	NA	87 (NA)	Only funnel plot (N)	No publication bias
Clements [1]	11(eCCA)	Case–control	Alcohol (drinker vs. non-drinker)	Random	OR	1.75	(1.20–2.55)	NA	87 (NA)	Only funnel plot (N)	No publication bias
*Cholecystolithiasis/gallbladder diease*											
Jiantao Wang [52]	18	Total case–control(10);Cohort(8)	Alcohol (highest vs. lowest)	Random	RR	0.62	(0.49–0.78)	NA	94.6 (0.000)	0.836	No publication bias
Jiantao Wang [52]#	10	Case–control(10)	Alcohol (highest vs. lowest)	Random	RR	0.58	(0.45–0.73)	NA	37.8 (0.107)	0.836	No publication bias
Jiantao Wang [52]#	8	Cohort(8)	Alcohol (highest vs. lowest)	Random	RR	0.66	(0.48–0.91)	NA	96.8 (0.000)	0.836	No publication bias
Jiantao Wang [52]	3	Case–control(1);Cohort(2)	Alcohol (types of drink beer highest vs. lowest)b	Random	RR	0.64	(0.52–0.78)	NA	0 (0.368)	0.836	No publication bias
Jiantao Wang [52]	3	Case–control(1);Cohort(2)	Alcohol (types of drink wine highest vs. lowest)b	Random	RR	0.72	(0.54–0.96)	NA	44.1 (0.167)	0.836	No publication bias
Jiantao Wang [52]	2	Cohort(2)	Alcohol (types of drink liquor highest vs. lowest)b	Random	RR	0.71	(0.64–0.85)	NA	1 (0.421)	0.836	No publication bias
Byung [27]	23	Case–control(14);Cohort(9)	Alcohol (drinker vs. non-drinker)	Random	RR	0.84	(0.79–0.89)	NA	61 (< 0.01)	0.009	Exist publication bias
Byung [27]	11	Total case–control(5);Cohort(6)	Alcohol (Light vs. none)^d^	Random	RR	0.96	(0.94–0.99)	NA	0 (0.75)	0.383	No publication bias
Byung [27]#	5	Case–control	Alcohol (Light vs. none)^d^	Random	RR	0.98	(0.95–1.01)	NA	0 (0.99)	0.383	No publication bias
Byung [27]#	6	Cohort	Alcohol (Light vs. none)^d^	Random	RR	0.94	(0.89–0.98)	NA	0 (0.51)	0.383	No publication bias
Byung [27]	14	Total case–control(8);Cohort(6)	Alcohol (Moderate vs. none)^d^	Random	RR	0.80	(0.75–0.85)	NA	17 (0.27)	0.523	No publication bias
Byung [27]#	8	Case–control	Alcohol (Moderate vs. none)^d^	Random	RR	0.76	(0.72–0.80)	NA	0 (0.70)	0.523	No publication bias
Byung [27]#	6	Cohort	Alcohol (Moderate vs. none)^d^	Random	RR	0.85	(0.80–0.91)	NA	0 (0.57)	0.523	No publication bias
Byung [27]	14	Total case–control(8);Cohort(6)	Alcohol (Heavy vs. none)^d^	Random	RR	0.66	(0.56–0.79)	NA	61 (< 0.01)	0.602	No publication bias
Byung [27]#	8	Case–control	Alcohol (Heavy vs. none)^d^	Random	RR	0.58	(0.40–0.85)	NA	54 (0.03)	0.602	No publication bias
Byung [27]#	6	Cohort	Alcohol (Heavy vs. none)^d^	Random	RR	0.73	(0.68–0.79)	NA	0 (0.61)	0.602	No publication bias

#: Subgroup analysis of the different study design types of the corresponding study

a: Alcoholic drinks per day(0 [referent], > 0–0.5, > 0.5–1, 1– < 3, 3– < 5, > 5 drink/d) and continuously (analyzed per one drink), One alcoholic drink was defined as 14 g of ethanol

b: The types of drink: wine, beer and liquor

c: The author decided to consider as light, moderate and heavy drinking every interval whose midpoint was respectively ≤ 12.5 g, ≤ 50 g and > 50 g per day of alcohol

d: Drinking level for each category: light, F < 7 and M < 14 g/day; moderate, F 7–17 and M 14–18 g/day; high, F > 14 and M > 28 g/day. F, female; M, male, B, both

### Gallbladder cancer

In the studies we included, there were some dietary factors, such as all spicy food [29], chili pepper [29] and tea [22] intake, which were not related to the risk of gallbladder cancer.

Type two diabetes mellitus was associated with an increased risk of gallbladder cancer (RR = 1.56, 95% CI 1.36–1.79) [41]. Compared with normal subjects, T2DM increased the risk of gallbladder cancer by 56%. Overweight and obesity are also risk factors for gallbladder cancer. Being overweight increased the risk by 17% (RR = 1.17, 95% CI 1.07–1.28), while in obesity this figure rose to 62% (RR = 1.62, 95% CI 1.49–1.75) [42]. These results are in good agreement with the results of subgroup analyses of different study models (case control and cohort studies) in the meta-analysis.

### Bile duct cancer

The consumption of tea was related to a reduced risk of bile duct cancer [36] (RR = 0.66,95% CI 0.48–0.85), although this was not apply to gallbladder cancer. According to dose–response analyses, the risk of bile duct cancer decreased by 4% with each additional cup of tea per day (RR = 0.96, 95% CI 0.93–0.98, *p* = 0.001). Due to the limited number of studies, no further study has investigated the effect of different types of tea intake on reducing the risk of bile duct cancer.

For fruit and vegetable consumption, we found that they have a significant effect on reducing the incidence of bile duct cancer [37]. In terms of vegetables consumption, the summary RR was 0.48 (95%CI 0.22–0.74). For dose–response analysis, every 100 g increment of vegetables consumed per day was associated with a 69 percent reduction in the risk of bile duct cancer (RR = 0.31, 95% CI 0.20–0.47, *p* < 0.001). In term of fruits consumption, the summary RR was 0.47 (95% CI 0.32–0.61) and the summary RR every 100 g increment a day was not statistically significant. Although the summary data show that vegetable and fruits consumption can reduce the risk of bile duct cancer, a cohort study in this meta-analysis showed that neither consumption were associated with a reduced risk of bile duct cancer. Therefore, the relationship between vegetable or fruit consumption and the risk of bile duct cancer remains to be further evaluated by larger and more comprehensive clinical studies.

In addition, some studies from Thailand have shown that eating raw fish [30] (RR = 2.54, 95% CI 1.94–3.35, *p* < 0.00001) and high-nitrate foods [23] (RR = 1.41, 95% CI 1.05–1.91, *p* = 0.024) increases the risk of bile duct cancer. Because these studies are meta-analyses of retrospective case–control studies and the sample size is small, the evidence they can provide is very limited. There was no obvious significant association of coffee [13], fermented fish [30], glutinous rice [30], meat [30], betel nut [30], fermented meats [23] and rice [23] consumption with bile duct cancer.

Similarly, some nutritional indicators are also associated with the risk of bile duct cancer. Both overweight and obesity increase the risk of eCCA [42]. Overweight increased the risk of eCCA by 26% (RR = 1.26, 95% CI 1.14–1.39), while obesity increased the risk by 48% (RR = 1.48, 95% CI 1.21–1.81). Although subgroup analysis of case–control studies showed that overweight was not associated with the risk of eCCA, we had more reason to believe the evidence provided by cohort studies. Obesity and diabetes are also risk factors for iCCA [24], in obese and diabetic subjects, the RR values of iCCA were 1.49 (95%CI 1.32–1.70, *p* < 0.001) and 1.53 (95%CI 1.31–1.78, *p* < 0.001) respectively.

### Cholecystolithiasis/gallbladder diseases

Since many studies did not separate cholecystolithiasis from other gallbladder diseases such as acute cholecystitis, some of the studies we included may not be limited to cholecystolithiasis.

Although studies have confirmed that coffee consumption has no obvious relationship with the incidence of biliary cancers, coffee consumption is a protective factor in the formation of gallstones [39]. Overall, the combined RR was 0.83 (95% CI 0.76–0.89), and each additional cup of coffee consumed per day was associated with a 5% (RR = 0.95, 95% CI 0.91–1.00, *p* = 0.049) reduction in cholecystolithiasis risk. As for the relationship between vegetable and fruit consumption and cholecystolithiasis, current evidence suggests that vegetable consumption is associated with a 17% (RR = 0.83, 95% CI 0.74–0.94) lower risk and fruit with a 12% (RR = 0.83, 95% CI 0.83–0.92) lower risk [40]. Moreover, there is a dose–response relationship between the intake of vegetables and fruits and the risk of disease, that is, for each additional 200 g per day, the corresponding risk of disease will be reduced by 4% (RR = 0.96, 95% CI 0.93–0.98, *p* = 0.001) and 3% (RR = 0.97, 95% CI 0.96–0.98, *p* = 0.001).

In addition, some nutritional indicators may be related to the occurrence of gallstone, such as Glycemic index rate [25] (RR = 1.26, 95% CI 1.13–1.40, *p* < 0.0001), Glycemic load rate [25] (RR = 1.41, 95% CI 1.25–1.60, *p* < 0.0001), diabetes mellitus [33] (RR = 1.41, 95% CI 1.56, 1.26–1.93), every 5 unit increment of BMI [34] (RR = 1.63, 95% CI 1.49–1.78), every 10 cm increment of waist circumference [34] (RR = 1.46, 95% CI 1.24–1.72), every 0.1 unit increment in waist-to-hip ratio [34] (RR = 1.44, 95% CI 1.26–1.64) and non-HDL cholesterol [26] (RR = 1.19, 95% CI 1.07–1.32).

### Alcohol consumption and biliary tract diseases

Our study included eight meta-analyses that discussed the relationship between alcohol consumption and biliary diseases.

In these studies, three meta-analyses reported the relationship between alcohol consumption and gallbladder cancer [21, 27, 31]. Bagnardi et al. [21] defined daily alcohol intake ≦12.5 g, ≦50 g and ≧50 g as light, moderate, and heavy alcohol consumption, respectively. Their meta analysis found that heavy drinking was associated with a marked increased risk of gallbladder cancer (RR = 2.64, 95% CI 1.62–4.30). The association between alcohol consumption and gallbladder cancer risk was not statistically significant for light (RR = 1.23, 95% CI 0.84–1.83) to moderate (RR = 0.88, 95% CI 0.68–1.13) drinkers. In a meta-analysis based on cohort studies, Emma E. McGee et al. [27] further divided the aggregated cohort studies into 6 subgroups (0,0–0.5,0.5–1,1–3,3–5, > 5 drink/d), based on drink/d (14 g of ethanol/d). The subgroups were compared with the 0 drink/d group and no statistical association was found between alcohol consumption and the risk of gallbladder cancer. There was no dose–response effect between alcohol consumption and gallbladder cancer, either. In another meta-analysis, Li et al. [31]. found a 30% reduction in gallbladder cancer among drinkers compared to non-drinkers (OR = 0.7, 99%CI 0.49–1.00, *p* = 0.009).

In the study of intrahepatic cholangiocarcinoma, Emma E. McGee et al. [27] found that the risk of iCCA was reduced in patients with > 0–0.5 drink/d compared with non-drinkers (HR = 0.79, 95%CI 0.62–1.00). When drinking > 5 drink/d, The risk of iCCA was 1.35 times higher than that of non-alcohol consumption (HR = 2.35, 95%CI 1.46–3.78), and there was a dose–response effect between the risk of iCCA and the amount of alcohol consumed (every 1drink/d increment, HR = 1.03, 95%CI 1.01–1.06, *p* = 0.04).Similarly, in the meta-analysis conducted by Clements et al. [1]., drinkers had an approximately 2.15-fold increased risk for iCCA (OR = 3.35, 95%CI 2.24–4.41) and a approximately 0.75-fold increased risk for eCCA (OR = 1.75, 95%CI 1.20–2.55) compared to non-drinkers.

As for cholecystolithiasis or gallbladder disease, although drinking is a risk factor for biliary cancers, Byung et al. [38]. found that drinking can reduce the risk of cholecystolithiasis by 16% (RR = 0.84, 95% CI = 0.79–0.89). At the same time, compared with non-drinkers, the risk of cholecystolithiasis decreased gradually with the increase of alcohol consumption. In mild, moderate and severe drinkers, the risk decreased by 4% (RR = 0.96, 95% CI = 0.94–0.99), 20% (RR = 0.80, 95% CI = 0.75–0.85) and 34% (RR = 0.66, 95% CI = 0.56–0.79) respectively. Similarly, we also found the same conclusion in the study of Jiantao Wang et al. [28] (Alcohol consumption highest vs. lowest, RR = 0.62, 95%CI 0.49–0.78). In addition, they also studied the consumption of different types of alcoholic beverages and the risk of cholecystolithiasis. The results showed that increased consumption of beer (RR = 0.64, 95%CI 0.52–0.78), wine (RR = 0.72, 95%CI 0.54–0.96), and liquor (RR = 0.71, 95%CI 0.64–0.85) tended to reduce the risk of gallstones.

### Heterogeneity, publication bias and small study effect

Of all the items we summarized (all items in Tables 2, 3 and 4), 52 items presented low heterogeneity (I^2^ < 25%); 36 items had moderate-to-high heterogeneity (25% < I^2^ < 75%), and 19 items had very high levels heterogeneity (I^2^ > 75%). In addition, there were 7 items that did not report heterogeneity. For evidence with significant heterogeneity (*p* < 0.05), the quality of evidence will be degraded in the evaluation of evidence quality.

This umbrella review used Egger’s test to summarize publication bias and small study effects in meta-analyses. Of the 24 meta-analyses, 7 studies did not measure publication bias, 3 reported significant publication bias, and the remaining did not report significant publication bias (Table 1).

### AMSTAR2 and GRADE classification

The methodological quality of the meta-analyses included in our study were assessed using AMSTAR2 scale, and the results of the review were rated as high, moderate, low, and critically low. Overall, the vast majority (21 studies, 87.5%) of methodological qualities of the meta-analyses were assessed as “critically low” by AMSTAR2 scale (Table 5). The remaining three meta-analyses were assessed as “low” and no one was assessed “moderate” or “high”.

**Table 5:** Methodological quality of the systematic review and meta-analyses were assessed using the AMSTAR2 scale

Study	Q1	Q2^*^	Q3	Q4^*^	Q5	Q6	Q7^*^	Q8	Q9^*^	Q10	Q11^*^	Q12	Q13^*^	Q14	Q15^*^	Q16	AMSTAR-2 overall quality
Bagnardi [21]	Y	N	N	Y	N	Y	N	PY	PY	N	Y	Y	Y	Y	N	Y	Critically low
Clements [1]	Y	N	N	Y	N	N	N	PY	PY	N	Y	N	N	N	Y	Y	Critically low
Godos [13]	Y	N	N	PY	Y	Y	Y	Y	PY	N	Y	N	Y	Y	Y	Y	Low
Chen [29]	Y	N	N	Y	Y	Y	N	Y	PY	N	Y	Y	Y	N	Y	Y	Critically low
Xiong [36]	Y	N	N	PY	N	Y	N	Y	Y	N	N	N	N	N	Y	Y	Critically low
ZHU [22]	Y	N	N	Y	N	Y	N	PY	PY	N	Y	N	N	Y	Y	Y	Critically low
Huai [37]	Y	N	N	Y	Y	Y	N	PY	PY	N	Y	N	Y	Y	Y	Y	Critically low
Kamsa-ard [30]	Y	N	N	N	Y	Y	N	N	PY	N	N	N	N	N	N	Y	Critically low
Steele [23]	Y	N	N	Y	N	Y	N	PY	N	N	N	N	Y	N	N	Y	Critically low
Daniel [26]	Y	Y	N	Y	Y	Y	N	Y	Y	N	N	N	N	N	N	Y	Critically low
Byung [38]	Y	N	Y	Y	Y	Y	N	PY	PY	N	N	N	Y	N	Y	Y	Critically low
Zhang [39]	Y	N	N	Y	Y	Y	N	Y	Y	N	Y	Y	Y	Y	Y	Y	Critically low
Zhang [40]	Y	N	N	Y	Y	Y	N	PY	Y	N	Y	Y	Y	Y	Y	Y	Critically low
Ying Li [31]	Y	N	N	Y	N	Y	N	PY	PY	N	Y	N	N	Y	N	Y	Critically low
Emma E. McGee [27]	Y	N	N	N	N	N	N	PY	PY	N	Y	N	N	N	N	Y	Critically low
Xiao-Hua Ye [35]	Y	N	N	Y	Y	Y	N	PY	PY	N	Y	Y	N	Y	Y	Y	Critically low
Jiantao Wang [28]	Y	N	N	Y	N	Y	N	PY	PY	N	Y	N	N	Y	Y	Y	Critically low
Gu [41]	Y	N	N	Y	Y	Y	N	Y	Y	N	Y	Y	Y	Y	Y	Y	Critically low
Li [42]	Y	N	N	Y	Y	N	N	Y	Y	N	N	Y	N	N	Y	Y	Critically low
Aune [33]	Y	N	N	Y	N	N	Y	Y	Y	N	Y	Y	Y	Y	Y	Y	Low
Barclay [25]	Y	N	N	Y	Y	Y	N	Y	N	N	N	Y	N	N	Y	Y	Critically low
Dagfinn Aune [34]	Y	N	Y	Y	N	N	Y	Y	Y	N	Y	Y	Y	Y	Y	Y	Low
Petrick [24]	Y	N	N	PY	Y	Y	N	Y	N	N	N	Y	Y	N	Y	Y	Critically low

AMSTAR-2 items: Q1: Did the research questions and inclusion criteria for the review include the components of PICO? Q2: Did the report of the review contain an explicit statement that the review methods were established prior to the conduct of the review, and did the report justify any signifificant deviations from the protocol? Q3: Did the review authors explain their selection of the study designs for inclusion in the review? Q4: Did the review authors use a comprehensive Literature search strategy? Q5: Did the review authors perform study selection in duplicate? Q6: Did the review authors perform data extraction in duplicate? Q7: Did the review authors provide a list of excluded studies and justify the exclusions? Q8: Did the review authors describe the included studies in adequate detail? Q9: Did the review authors use a satisfactory technique for assessing the risk of bias (RoB) in individual studies that were included in the review? Q10: Did the review authors report on the sources of funding for the studies included in the review? Q11: If meta-analysis was performed, did the review authors use appropriate methods for statistical combination of results? Q12: If meta-analysis was performed, did the review authors assess the potential impact of RoB in individual studies on the results of the meta-analysis or other evidence synthesis? Q13: Did the review authors account for RoB in primary studies when interpreting/discussing the results of the review? Q14: Did the review authors provide a satisfactory explanation for, and discussion of, any heterogeneity observed in the results of the review? Q15: If they performed quantitative synthesis, did the review authors carry out an adequate investigation of publication bias (small study bias) and discuss its likely impact on the results of the review? Q16: Did the review authors report any potential sources of conflflict of interest, including any funding they received for conducting the review?

Since the studies we included were all meta-analyses based on retrospective studies, all evidence was first lowered by two grades, from high level to low level during GRADE evaluation. Next, we decided whether to continue to downgrade the evidence according to whether there was risk of bias, indirectness, inconsistency, imprecision and publication bias. After checking whether there were large effects, dose–response gradients and plausible confounding of evidence, whether to upgrade the level of evidence and finally determine the strength of each evidence level was decided.

In this umbrella review, we summarized 83 independent outcomes (Table 5). Regarding the risk of bias, 44 outcomes were downgraded due to inadequate control for confounding factors (including inaccuracy in measuring all known prognostic factors; Prognostic factors were not matched and/or not adjusted in the statistical analysis). We judged the imprecision of evidence by the 95% confidence interval of each evidence and the optimal information size (OIS). If the sample size of evidence was lower than the OIS standard, the confidence intervals contained invalid values, or the confidence intervals did not exclude significant benefits or harms (95%CI contained 1, with lower limit < 0.75, upper limit > 1.25), the quality level of evidence would be reduced. We found a total of 38 outcomes of Imprecision and downgraded one level. The inconsistency and publication bias were mainly evaluated according to the *I*^2^ and Egger’s test of meta-analyses included in our study. In our study, 35 outcomes were downgraded due to inconsistencies and 45 outcomes were downgraded due to suspected publication bias. None of the outcomes was downgraded due to indirectness. In terms of upgrading factors, seven of the outcomes were upgraded due to large effect (Relative effect > 2 or < 0.5), 14 due to dose response gradient, and 0 due to plausible confounding.

In short, among 83 independent outcomes, 5 were rated as moderate, 16 as low, and the rest as very low (Table 6).

**Table 6:** AMSTAR2 and GRADE classification of the evidence

Summary of findings					Certainty assessment(degradation factor)					Certainty assessment (Escalation factors)			Importance	Grade	AMSTAR2
First author, Year	Dietary and nutrition related factor	Study design(number)	Outcome	Relative effect (95% CI)	Risk of bias	Inconsistency	Indirectness	Imprecision	Publication bias	Large effect	Plausible confounding	Dose response gradient			
Chen [19]	All spicy food	Case–control(6)	Gallbladder cancer	OR 1.78 (0.83–3.83)	Serious^e^	Serious^f^	Not serious	Serious^g^	Undetected	No	No	No	6-Important	⨁◯◯◯ Very low	Critically low
Chen [19]	Chili pepper	Case–control(6)	Gallbladder cancer	OR 1.78 (0.83–3.83)	Serious^e^	Serious^f^	Not serious	Serious^g^	Undetected	No	No	No	6-Important	⨁◯◯◯ Very low	Critically low
ZHU [20]	Tea	Case–control(4);Cohort(2)	Gallbladder cancer	OR 0.67 (0.40–1.12)	Serious^e^	Serious^f^	Not serious	Serious^g^	Undetected	No	No	No	6-Important	⨁◯◯◯ Very low	Critically low
ZHU [20]	Tea (highest vs. lowest/none)	Case–control(3);Cohort(1)	Gallbladder cancer	OR 0.57 (0.25–1.29)	Serious^e^	Serious^f^	Not serious	Serious^g^	Undetected	No	No	No	6-Important	⨁◯◯◯ Very low	Critically low
ZHU [20]	Tea (moderate vs. low/none)	Case–control(3);Cohort(1)	Gallbladder cancer	OR 0.62 (0.33–1.14)	Serious^e^	Serious^f^	Not serious	Serious^g^	Undetected	No	No	No	6-Important	⨁◯◯◯ Very low	Critically low
Godos [8]	Coffee	Cohort(5);Case–control(3)	Biliary tract cancer	OR 0.83 (0.64–1.08)	Not serious	Not serious	Not serious	Serious^g^	Undetected	No	No	No	6-Important	⨁◯◯◯ Very low	Low
Xiong [21]	Tea	Case–control(5);Cohort(3)	Biliary tract cancer	RR 0.66 (0.48–0.85)	Serious^e^	Serious^f^	Not serious	Not serious	Undetected	No	No	Yes	7-Critical	⨁◯◯◯ Very low	Critically low
Xiong [21]	Tea (every 1cup/day increment)	Case–control(5);Cohort(3)	Biliary tract cancer	RR 0.96 (0.93–0.98)	Serious^e^	Serious^f^	Not serious	Not serious	Undetected	No	No	Yes	7-Critical	⨁◯◯◯ Very low	Critically low
Huai [22]	Vegetable	Case–control(8);Cohort(1); Nested case–control(1)	Biliary tract cancer	RR 0.48 (0.22–0.74)	Not serious	Serious^f^	Not serious	Not serious	Undetected	Yes	No	Yes	7-Critical	⨁⨁⨁◯ Moderate	Critically low
Huai [22]	Vegetable (every 100 g/day increment)	Case–control(6);Cohort(1); Nested case–control(1)	Biliary tract cancer	RR 0.31 (0.20–0.47)	Not serious	Serious^f^	Not serious	Not serious	Undetected	Yes	No	Yes	7-Critical	⨁⨁⨁◯ Moderate	Critically low
Huai [22]	Fruit	Case–control(11);Cohort(1); Nested case–control(1)	Biliary tract cancer	RR 0.47 (0.32–0.61)	Not serious	Serious^f^	Not serious	Not serious	Undetected	Yes	No	No	7-Critical	⨁⨁◯◯ Low	Critically low
Huai [22]	Fruit (every 100 g/day increment)	Case–control(6);Cohort(1); Nested case–control(1)	Biliary tract cancer	RR 0.89 (0.66–1.18)	Not serious	Serious^f^	Not serious	Serious^g^	Undetected	No	No	Yes	6-Important	⨁◯◯◯ Very low	Critically low
Kamsa-ard [23]	Raw Fish	Case–control(3)	Biliary tract cancer	OR 2.54 (1.94–3.35)	Serious^e^	Not serious	Not serious	Not serious	Strongly suspected	Yes	No	No	7-Critical	⨁◯◯◯ Very low	Critically low
Kamsa-ard [23]	Fermented Fish	Case–control(2)	Biliary tract cancer	OR 1.61 (0.76–3.41)	Serious^e^	Not serious	Not serious	Serious^g^	Strongly suspected	No	No	No	6-Important	⨁◯◯◯ Very low	Critically low
Kamsa-ard [23]	Glutinous Rice	Case–control(3)	Biliary tract cancer	OR 1.3 (0.85–2.01)	Serious^e^	Not serious	Not serious	Serious^g^	Strongly suspected	No	No	No	6-Important	⨁◯◯◯ Very low	Critically low
Kamsa-ard [23]	Meat	Case–control(2)	Biliary tract cancer	OR 1.03 (0.57–1.85)	Serious^e^	Not serious	Not serious	Serious^g^	Strongly suspected	No	No	No	6-Important	⨁◯◯◯ Very low	Critically low
Kamsa-ard [23]	Betel nut	Case–control(3)	Biliary tract cancer	OR 1.45 (0.69–3.02)	Serious^e^	Not serious	Not serious	Serious^g^	Strongly suspected	No	No	No	6-Important	⨁◯◯◯ Very low	Critically low
Steele [24]	Fermented Meats	Total case–control(2) @Nested case–control(1)	Biliary tract cancer	OR 1.81 (0.96–3.39)	Serious^e^	Not serious	Not serious	Serious^g^	Strongly suspected	No	No	No	6-Important	⨁◯◯◯ Very low	Critically low
Steele [24]	High Nitrate Foods	Total case–control(3) @Nested case–control(2)	Biliary tract cancer	OR 1.41 (1.05–1.91)	Serious^e^	Serious^f^	Not serious	Not serious	Strongly suspected	No	No	No	7-Critical	⨁◯◯◯ Very low	Critically low
Steele [24]	Rice	Case–control(2)	Biliary tract cancer	OR 0.88 (0.48–1.63)	Serious^e^	Not serious	Not serious	Serious^g^	Strongly suspected	No	No	No	6-Important	⨁◯◯◯ Very low	Critically low
Zhang [28]	Coffee	Cohort(7)	Cholecystolithiasis/gallbladder diease	RR 0.83 (0.76–0.89)	Not serious	Not serious	Not serious	Not serious	Undetected	No	No	Yes	7-Critical	⨁⨁⨁◯ Moderate	Critically low
Zhang [28]	Coffee (every 1Cup/Day increment)	Cohort(4)	Cholecystolithiasis/gallbladder diease	RR 0.95 (0.91–1.00)	Not serious	Serious^f^	Not serious	Not serious	Undetected	No	No	Yes	7-Critical	⨁⨁◯◯ Low	Critically low
Zhang [29]	Vegetables	Case–control(4);Cohort(9); @Cross sectional(1)	Cholecystolithiasis/gallbladder diease	RR 0.83 (0.74–0.94)	Not serious	Serious^f^	Not serious	Not serious	Undetected	No	No	Yes	7-Critical	⨁⨁◯◯ Low	Critically low
Zhang [29]	Vegetables(every 200 g/Day increment)	Cohort(6)	Cholecystolithiasis/gallbladder diease	RR 0.96 (0.93–0.98)	Not serious	Serious^f^	Not serious	Not serious	Undetected	No	No	Yes	7-Critical	⨁⨁◯◯ Low	Critically low
Zhang [29]	Fruits	Cohort(5)	Cholecystolithiasis/gallbladder diease	RR 0.88 (0.83–0.92)	Not serious	Not serious	Not serious	Not serious	Undetected	No	No	Yes	7-Critical	⨁⨁⨁◯ Moderate	Critically low
Zhang [29]	Fruits (every 200 g/Day increment)	Cohort(4)	Cholecystolithiasis/gallbladder diease	RR 0.97 (0.96–0.98)	Not serious	Not serious	Not serious	Not serious	Undetected	No	No	Yes	7-Critical	⨁⨁⨁◯ Moderate	Critically low
Gu [17]	Type 2 DM	Case–control(8); Cohort(12)	Gallbladder cancer	RR 1.56 (1.36–1.79)	Not serious	Serious^f^	Not serious	Not serious	Strongly suspected	No	No	No	7-Critical	⨁◯◯◯ Very low	Critically low
Li [18]	Overweight	Case–control(8); Cohort(9)	Gallbladder cancer	RR 1.17 (1.07–1.28)	Not serious	Serious^f^	Not serious	Not serious	Undetected	No	No	No	7-Critical	⨁◯◯◯ Very low	Critically low
Li [18]	Obesity	Case–control(9); Cohort(13)	Gallbladder cancer	RR 1.62 (1.49–1.75)	Not serious	Not serious	Not serious	Not serious	Undetected	No	No	No	7-Critical	⨁⨁◯◯ Low	Critically low
Li [18]	Overweight	Case–control(4); Cohort(4)	Biliary tract cancer-eCCA	RR 1.26 (1.14–1.39)	Not serious	Not serious	Not serious	Not serious	Undetected	No	No	No	7-Critical	⨁⨁◯◯ Low	Critically low
Li [18]	Obesity	Case–control(9); Cohort(7)	Biliary tract cancer-eCCA	RR 1.48 (1.21–1.81)	Not serious	Serious^f^	Not serious	Not serious	Undetected	No	No	No	7-Critical	⨁◯◯◯ Very low	Critically low
Petrick [25]	Obesity	Nested case–control(3); Cohort(1)	Biliary tract cancer-iCCA	RR 1.49 (1.32–1.70)	Not serious	Not serious	Not serious	Not serious	Undetected	No	No	No	7-Critical	⨁⨁◯◯ Low	Critically low
Petrick [25]	DM	Nested case–control(4); Cohort(2)	Biliary tract cancer-iCCA	RR 1.53 (1.31–1.78)	Not serious	Serious^f^	Not serious	Not serious	Undetected	No	No	No	7-Critical	⨁◯◯◯ Very low	Critically low
Aune [31]	DM	Cohort(10)	Cholecystolithiasis/gallbladder diease	RR 1.56 (1.26–1.93)	Not serious	Serious^f^	Not serious	Not serious	Undetected	No	No	No	7-Critical	⨁◯◯◯ Very low	Low
Barclay [30]	Glycemic index rate (highest vs. lowest)	Cohort(2)	Cholecystolithiasis/gallbladder diease	RR 1.26 (1.13–1.40)	Serious^e^	Serious^f^	Not serious	Not serious	Undetected	No	No	No	7-Critical	⨁◯◯◯ Very low	Critically low
Barclay [30]	Glycemic load rate (highest vs. lowest)	Cohort(2)	Cholecystolithiasis/gallbladder diease	RR 1.41 (1.25–1.60)	Serious^e^	Serious^f^	Not serious	Not serious	Undetected	No	No	No	7-Critical	⨁◯◯◯ Very low	Critically low
Dagfinn Aune [32]	Every 5 unit increment of BMI	Cohort(17)	Cholecystolithiasis/gallbladder diease	RR 1.63 (1.49–1.78)	Not serious	Serious^f^	Not serious	Not serious	Undetected	No	No	Yes	7-Critical	⨁⨁◯◯ Low	Low
Dagfinn Aune [32]	Every 10 cm increment of waist circumference	Cohort(5)	Cholecystolithiasis/gallbladder diease	RR 1.46 (1.24–1.72)	Not serious	Serious^f^	Not serious	Not serious	Strongly suspected	No	No	Yes	7-Critical	⨁◯◯◯ Very low	Low
Dagfinn Aune [32]	Every 0.1 unit increment in waist-to-hip ratio	Cohort(4)	Cholecystolithiasis/gallbladder diease	RR 1.44 (1.26–1.64)	Not serious	Serious^f^	Not serious	Not serious	Strongly suspected	No	No	Yes	7-Critical	⨁◯◯◯ Very low	Low
Daniel [33]	Triglycerides	Cohort(2)	Cholecystolithiasis/gallbladder diease	OR 1.1 (0.99–1.22)	Not serious	Not serious	Not serious	Not serious	Strongly suspected	No	No	No	6-Important	⨁◯◯◯ Very low	Critically low
Daniel [33]	HDL cholesterol	Cohort(2)	Cholecystolithiasis/gallbladder diease	OR 0.87 (0.62–1.23)	Not serious	Not serious	Not serious	Serious^g^	Strongly suspected	No	No	No	6-Important	⨁◯◯◯ Very low	Critically low
Daniel [33]	Non-HDL cholesterol	Cohort(2)	Cholecystolithiasis/gallbladder diease	OR 1.19 (1.07–1.32)	Not serious	Serious^f^	Not serious	Not serious	Strongly suspected	No	No	No	7-Critical	⨁◯◯◯ Very low	Critically low
Ying Li [50]	Alcohol(drinker vs. non-drinker)	Case–control(2)	Gallbladder cancer	OR 0.7 @99%CI(0.49–1.00)	Serious^e^	Not serious	Not serious	Not serious	Strongly suspected	No	No	No	7-Critical	⨁◯◯◯ Very low	Critically low
Emma E. McGee, 2019 [35]	Alcohol(>0–0.5 vs. 0 drink/d)^a^	Cohort(26)	Gallbladder cancer	HR 1.07 (0.91–1.26)	Serious^e^	Not serious	Not serious	Serious^g^	Strongly suspected	No	No	No	6-Important	⨁◯◯◯ Very low	Critically low
Emma E. McGee, 2019 [35]	Alcohol(>0.5–1 vs. 0 drink/d)^a^	Cohort(26)	Gallbladder cancer	HR 1.1 (0.87–1.39)	Serious^e^	Not serious	Not serious	Serious^g^	Strongly suspected	No	No	No	6-Important	⨁◯◯◯ Very low	Critically low
Emma E. McGee [35]	Alcohol(1–<3 vs. 0 drink/d)^a^	Cohort(26)	Gallbladder cancer	HR 0.94 (0.74–1.21)	Serious^e^	Not serious	Not serious	Serious^g^	Strongly suspected	No	No	No	6-Important	⨁◯◯◯ Very low	Critically low
Emma E. McGee [35]	Alcohol(3–<5, vs. 0 drink/d)^a^	Cohort(26)	Gallbladder cancer	HR 1.16 (0.69–1.94)	Serious^e^	Not serious	Not serious	Serious^g^	Strongly suspected	No	No	No	6-Important	⨁◯◯◯ Very low	Critically low
Emma E. McGee [35]	Alcohol(>5 vs. 0 drink/d)^a^	Cohort(26)	Gallbladder cancer	HR 2.39 (0.63–9.12)	Serious^e^	Serious^f^	Not serious	Serious^g^	Strongly suspected	No	No	No	6-Important	⨁◯◯◯ Very low	Critically low
Emma E. McGee [35]	Alcohol(every 1drink/d increment)^a^	Cohort(26)	Gallbladder cancer	HR 0.98 (0.92–1.05)	Serious^e^	Not serious	Not serious	Not serious	Strongly suspected	No	No	No	6-Important	⨁◯◯◯ Very low	Critically low
Bagnardi [16]	Alcohol (Light vs. none)^c^	Case–control(4);Cohort(4)	Gallbladder cancer	RR 1.23 (0.84 − 1.83)	Not serious	Not serious	Not serious	Serious^g^	Strongly suspected	No	No	No	6-Important	⨁◯◯◯ Very low	Critically low
Bagnardi [16]	Alcohol (Moderate vs. none)^c^	Case–control(4);Cohort(4)	Gallbladder cancer	RR 0.88 (0.68 − 1.13)	Not serious	Not serious	Not serious	Serious^g^	Strongly suspected	No	No	No	6-Important	⨁◯◯◯ Very low	Critically low
Bagnardi [16]	Alcohol (Heavy vs. none)^c^	Case–control(4);Cohort(4)	Gallbladder cancer	RR 2.64 (1.62 − 4.30)	Not serious	Not serious	Not serious	Not serious	Strongly suspected	Yes	No	No	7-Critical	⨁⨁◯◯ Low	Critically low
Ying Li [50]	Alcohol(drinker vs. non-drinker)	Case–control(2)	Biliary tract cancer-eCCA	OR 1.14 99%CI(0.75–1.75)	Serious^e^	Not serious	Not serious	Serious^g^	Strongly suspected	No	No	No	6-Important	⨁◯◯◯ Very low	Critically low
Ying Li [50]	Alcohol(drinker vs. non-drinker)	Case–control(2)	Biliary tract cancer-VPC	OR 0.68 99%CI(0.20–2.37)	Serious^e^	Serious^f^	Not serious	Serious^g^	Strongly suspected	No	No	No	6-Important	⨁◯◯◯ Very low	Critically low
Emma E. McGee [35]	Alcohol(> 0–0.5 vs. 0 drink/d)^a^	Cohort(26)	Biliary tract cancer	HR 0.79 (0.62–1.00)	Serious^e^	Not serious	Not serious	Serious^g^	Strongly suspected	No	No	No	7-Critical	⨁◯◯◯ Very low	Critically low
Emma E. McGee [35]	Alcohol(> 0.5–1 vs. 0 drink/d)^a^	Cohort(26)	Biliary tract cancer	HR 0.91 (0.65–1.26)	Serious^e^	Not serious	Not serious	Serious^g^	Strongly suspected	No	No	No	6-Important	⨁◯◯◯ Very low	Critically low
Emma E. McGee [35]	Alcohol(1– < 3 vs. 0 drink/d)^a^	Cohort(26)	Biliary tract cancer	HR 0.98 (0.73–1.31)	Serious^e^	Not serious	Not serious	Serious^g^	Strongly suspected	No	No	No	6-Important	⨁◯◯◯ Very low	Critically low
Emma E. McGee [35]	Alcohol(3– < 5, vs. 0 drink/d)^a^	Cohort(26)	Biliary tract cancer	HR 1.25 (0.77–2.02)	Serious^e^	Not serious	Not serious	Serious^g^	Strongly suspected	No	No	No	6-Important	⨁◯◯◯ Very low	Critically low
Emma E. McGee [35]	Alcohol(> 5 vs. 0 drink/d)^a^	Cohort(26)	Biliary tract cancer	HR 2.35 (1.46–3.78)	Serious^e^	Not serious	Not serious	Not serious	Strongly suspected	Yes	No	No	7-Critical	⨁◯◯◯ Very low	Critically low
Emma E. McGee [35]	Alcohol(every 1drink/d increment)^a^	Cohort(26)	Biliary tract cancer	HR 1.03 (1.01–1.06)	Serious^e^	Not serious	Not serious	Not serious	Strongly suspected	No	No	No	7-Critical	⨁◯◯◯ Very low	Critically low
Emma E. McGee [35]	Alcohol(> 0–0.5 vs. 0 drink/d)^a^	Cohort(26)	Biliary tract cancer	HR 0.87 (0.68–1.12)	Serious^e^	Not serious	Not serious	Serious^g^	Strongly suspected	No	No	No	6-Important	⨁◯◯◯ Very low	Critically low
Emma E. McGee [35]	Alcohol(> 0.5–1 vs. 0 drink/d)^a^	Cohort(26)	Biliary tract cancer	HR 1.14 (0.82–1.58)	Serious^e^	Not serious	Not serious	Serious^g^	Strongly suspected	No	No	No	6-Important	⨁◯◯◯ Very low	Critically low
Emma E. McGee [35]	Alcohol(1– < 3 vs. 0 drink/d)^a^	Cohort(26)	Biliary tract cancer	HR 1.08 (0.74–1.58)	Serious^e^	Serious^f^	Not serious	Serious^g^	Strongly suspected	No	No	No	6-Important	⨁◯◯◯ Very low	Critically low
Emma E. McGee [35]	Alcohol(3– < 5, vs. 0 drink/d)^a^	Cohort(26)	Biliary tract cancer	HR 1.82 (0.98–3.39)	Serious^e^	Serious^f^	Not serious	Serious^g^	Strongly suspected	No	No	No	6-Important	⨁◯◯◯ Very low	Critically low
Emma E. McGee [35]	Alcohol(> 5 vs. 0 drink/d)^a^	Cohort(26)	Biliary tract cancer	HR 1.02 (0.64–1.62)	Serious^e^	Not serious	Not serious	Serious^g^	Strongly suspected	No	No	No	6-Important	⨁◯◯◯ Very low	Critically low
Emma E. McGee [35]	Alcohol(every 1drink/d increment)^b^	Cohort(26)	Biliary tract cancer	HR 1.03 (0.98–1.08)	Serious^e^	Not serious	Not serious	Not serious	Strongly suspected	No	No	No	6-Important	⨁◯◯◯ Very low	Critically low
Emma E. McGee [35]	Alcohol(> 0–0.5 vs. 0 drink/d)^a^	Cohort(26)	Biliary tract cancer	HR 1.08 (0.80–1.45)	Serious^e^	Not serious	Not serious	Serious^g^	Strongly suspected	No	No	No	6-Important	⨁◯◯◯ Very low	Critically low
Emma E. McGee [35]	Alcohol(> 0.5–1 vs. 0 drink/d)^a^	Cohort(26)	Biliary tract cancer	HR 0.99 (0.69–1.41)	Serious^e^	Not serious	Not serious	Serious^g^	Strongly suspected	No	No	No	6-Important	⨁◯◯◯ Very low	Critically low
Emma E. McGee [35]	Alcohol(1– < 3 vs. 0 drink/d)^a^	Cohort(26)	Biliary tract cancer	HR 1.33 (0.99–1.80)	Serious^e^	Not serious	Not serious	Serious^g^	Strongly suspected	No	No	No	6-Important	⨁◯◯◯ Very low	Critically low
Emma E. McGee [35]	Alcohol(3– < 5, vs. 0 drink/d)^a^	Cohort(26)	Biliary tract cancer	HR 1.16 (0.66–2.01)	Serious^e^	Not serious	Not serious	Serious^g^	Strongly suspected	No	No	No	6-Important	⨁◯◯◯ Very low	Critically low
Emma E. McGee [35]	Alcohol(> 5 vs. 0 drink/d)^a^	Cohort(26)	Biliary tract cancer	HR 1.59 (0.85–2.98)	Serious^e^	Not serious	Not serious	Serious^g^	Strongly suspected	No	No	No	6-Important	⨁◯◯◯ Very low	Critically low
Emma E. McGee [35]	Alcohol(every 1drink/d increment)^a^	Cohort(26)	Biliary tract cancer	HR 1 (0.95–1.04)	Serious^e^	Not serious	Not serious	Not serious	Strongly suspected	No	No	No	6-Important	⨁◯◯◯ Very low	Critically low
Xiao-Hua Ye [51]	Alcohol(drinker vs. non-drinker)	Case–control(6);Cohort(1)	Biliary tract cancer-eCCA	RR 1.09 (0.87–1.37)	Not serious	Not serious	Not serious	Serious^g^	Undetected	No	No	No	6-Important	⨁⨁◯◯ Low	Critically low
Clements [1]	Alcohol(drinker vs. non-drinker)	Case–control(15)	Biliary tract cancer-iCCA	OR 3.15 (2.24–4.41)	Not serious	Serious^f^	Not serious	Not serious	Undetected	Yes	No	No	7-Critical	⨁⨁◯◯ Low	Critically low
Clements [1]	Alcohol(drinker vs. non-drinker)	Case–control(11)	Biliary tract cancer-eCCA	OR 1.75 (1.20–2.55)	Not serious	Serious^f^	Not serious	Not serious	Undetected	No	No	No	7-Critical	⨁◯◯◯ Very low	Critically low
Jiantao Wang [52]	Alcohol(highest vs. lowest)	Case–control(10);Cohort(8)	Cholecystolithiasis/gallbladder diease	RR 0.62 (0.49–0.78)	Not serious	Serious^f^	Not serious	Not serious	Undetected	No	No	No	7-Critical	⨁◯◯◯ Very low	Critically low
Jiantao Wang	Alcohol(types of drink beer highest vs. lowest)^b^	Case–control(1); Cohort(2)	Cholecystolithiasis/gallbladder diease	RR 0.64 (0.52–0.78)	Not serious	Not serious	Not serious	Not serious	Undetected	No	No	No	7-Critical	⨁⨁◯◯ Low	Critically low
Jiantao Wang [52]	Alcohol(types of drink wine highest vs. lowest)^b^	Case–control(1); Cohort(2)	Cholecystolithiasis/gallbladder diease	RR 0.72 (0.54–0.96)	Not serious	Not serious	Not serious	Not serious	Undetected	No	No	No	7-Critical	⨁⨁◯◯ Low	Critically low
Jiantao Wang [52]	Alcohol(types of drink liquor highest vs. lowest)^b^	Cohort(2)	Cholecystolithiasis/gallbladder diease	RR 0.71 (0.64–0.85)	Not serious	Not serious	Not serious	Not serious	Undetected	No	No	No	7-Critical	⨁⨁◯◯ Low	Critically low
Byung [27]	Alcohol (drinker vs. non-drinker)	Case–control(14);Cohort(9)	Cholecystolithiasis/gallbladder diease	RR 0.84 (0.79–0.89)	Not serious	Serious^f^	Not serious	Not serious	Strongly suspected	No	No	No	7-Critical	⨁◯◯◯ Very low	Critically low
Byung [27]	Alcohol (Light vs. none)^d^	Case–control(5);Cohort(6)	Cholecystolithiasis/gallbladder diease	RR 0.96 (0.94–0.99)	Not serious	Not serious	Not serious	Not serious	Undetected	No	No	No	7-Critical	⨁⨁◯◯ Low	Critically low
Byung [27]	Alcohol (Moderate vs. none)^d^	Case–control(8);Cohort(6)	Cholecystolithiasis/gallbladder diease	RR 0.8 (0.75–0.85)	Not serious	Not serious	Not serious	Not serious	Undetected	No	No	No	7-Critical	⨁⨁◯◯ Low	Critically low
Byung [27]	Alcohol (Heavy vs. none)^d^	Case–control(8);Cohort(6)	Cholecystolithiasis/gallbladder diease	RR 0.66 (0.56–0.79)	Not serious	Serious^f^	Not serious	Not serious	Undetected	No	No	No	7-Critical	⨁◯◯◯ Very low	Critically low

a: Alcoholic drinks per day(0 [referent], > 0–0.5, > 0.5–1, 1– < 3, 3– < 5, > 5 drink/d) and continuously (analyzed per one drink), One alcoholic drink was defined as 14 g of ethanol

b: The types of drink: wine, beer and liquor

c: The author decided to consider as light, moderate and heavy drinking every interval whose midpoint was respectively ≤ 12.5 g, ≤ 50 g and > 50 g per day of alcohol

d: Drinking level for each category: light, F < 7 and M < 14 g/day; moderate, F 7–17 and M 14–18 g/day; high, F > 14 and M > 28 g/day. F, female; M, male, B, both

e: Failure to adequately control for confounding

f: Conclusions significant heterogeneity was reported

g:The credible interval contains invalid values and the credible interval does not exclude significant benefits or harms

## Discussion

### Main findings and interpretation of evidence

To promote the general population’s understanding of the impact of dietary and nutritional indicators on biliary disease risk, our study provide a comprehensive overview of the reported associations between diet and nutrition-related factors and biliary disease risk by incorporating evidence from existing systematic reviews and meta-analyses. Overall, we included 24 articles that included 83 risk estimates of dietary and nutrition-related factors associated with the incidence of gallbladder cancer, bile duct cancer, and gallstones. There was no high evidence to support an association among all the evidence evaluated. Only 5 associations were supported by moderate evidence and 16 associations were supported by low evidence.

In this umbrella review, the evaluation tools we used include AMSTAR2 and GRADE. The methodological quality of the meta-analyses included in this umbrella review was assessed by AMSTAR2. It mainly includes the following aspects of evaluation: research questions, inclusion standard PICO elements, system review plan, included study design type, literature search strategy, literature screening, data extraction, exclusion of specific details of literature, assessment of bias risk, assessment of the rationality of statistical analysis, assessment of the accuracy of interpretation of results, and assessment of financial support and conflict of interest. Based on the evaluation of the above projects, the results of the review is rated as high, moderate, low, and critically low [17]. The application of GRADE in the systematic reviews and meta analyses is to analyze the quality of the evidence, that is, to what extent the authenticity of the prognostic outcome can be assured. By examining five demotion factors, including risk of bias, Indirectness, inconsistency, imprecision and publication bias, three upgrade factors, namely large effect, dose–response gradient and plausible confounding, we divided the quality of evidence of systematic evaluation into four grades: high, moderate, low and very low [18, 19].

Based on available evidence, our study did not found that food or nutrition consumption (except alcohol consumption) was associated with increased risk of gallbladder cancer. Our study found that overweight, obesity and diabetes can increase the risk of gallbladder cancer, but the quality of evidence was rated low or very low. That doesn’t mean the conclusion is wrong. At present, existing studies have proposed the biological pathogenesis of gallbladder cancer caused by the above factors. It is generally believed that overweight and obesity contribute to gallbladder cancer by interfering with the metabolism of lipids and endogenous hormones, affecting the movement of the gallbladder and increasing the risk of gallstones [43]. Other studies also believe that obesity will inevitably increase the accumulation of fat in the gallbladder, leading to fatty gallbladder disease and aggravating local inflammation, which is also an important mechanism to promote the occurrence of gallbladder cancer [44]. In type 2 diabetes, the possible mechanisms contributing to gallbladder cancer include: Hyperinsulinemia and up-regulation of insulin-like growth factor-1 (IGF-1) levels promote cell proliferation and inhibit apoptosis. Hyperglycemia stimulates tumor growth by inducing the increase of insulin and IGF-1 levels. In addition, some studies have suggested that other dietary factors, such as the consumption of green onions, seaweed and kelp, are negatively correlated with gallbladder and bile duct cancer, while pickled vegetables and meats are positively correlated [45]. These studies were not included in this umbrella review because there was no meta-analysis to evaluate these results.

As for bile duct cancer, it is important to note that in bile duct cancer studies, some have included gallbladder and bile duct cancer together, or have not performed a subgroup analysis by bile duct cancer type. Subgroup analysis were not reworked because of limited data availability. Our study found that drinking tea is a protective factor of cholangiocarcinoma [36]. In terms of the biological mechanism by which tea drinking can reduce the risk of cancer, studies have confirmed that tea contains a large amount of tea polyphenols, which can inhibit cell proliferation, enhance apoptosis, inhibit cell invasion, angiogenesis and metastasis by inhibiting enzyme activity and signal transduction pathway [46]. Although the level of this evidence is very low, it still has certain suggestive significance. We observed a strong inverse association between fruit and vegetable consumption and bile duct cancer incidence [37]. Two outcomes related to vegetable consumption were rated as moderate, and two outcomes related to fruit consumption were rated as low and very low respectively. Fruits and vegetables are not only high in fiber, but also have anti-tumor properties of micronutrients and macronutrients; As such, they are reasonable targets for dietary prevention. In two meta-analyses from Thailand [23, 30], raw fish and high nitrate food consumption were suspected risk factors for bile duct cancer. Because the studies included in these two meta-analyses were limited to Thailand, the possibility of publication bias were high and the number of cases included was small, the conclusions of these studies were relatively limited. Among the nutrition-related indicators associated with bile duct cancer, similar to gallbladder cancer, higher body mass index and diabetes still increased the risk of bile duct cancer. Furthermore, other studies have shown that some specific dietary patterns can also affect the occurrence of bile duct cancer. For example, in a cohort study [10], a Mediterranean (MED) diet and the Dietary Approaches to Stop Hypertension (DASH) significantly reduced the risk of bile duct cancer. Similarly, this research was not included in this umbrella review because it was not further studied by evidence-based medicine.

Cholelithiasis or gallbladder diease is also one of the most common diseases of the biliary system. There has been clear epidemiological evidence that gallstone is a risk factor for gallbladder cancer [47]. Therefore, the research on the risk factors of gallstone is of great significance both from the perspective of prevention of gallbladder cancer and health economics. In our study, we found 3 moderate intensity outcomes, 4 low intensity outcomes, and the other outcomes levels were very low. Based on the available evidence, we recommend proper intake of coffee, fruits and vegetables to reduce the risk of gallstones. In the evidence of nutrition related indicators we included, almost all the evidence related to cholelithiasis (blood glucose related indicators and BMI related indicators) were consistent with the corresponding evidence of gallbladder cancer or cholangiocarcinoma, but the level of all evidence were not high. Recent studies have pointed out that high fructose, low fiber, high fat and low vitamin C will increase the risk of gallstone formation. On the other hand, a high intake of monounsaturated fats and fiber, moderate intake of olive oil, fish, plant proteins, fruit, coffee, and vitamin C supplementation were all protective [48]. Furthermore, different dietary patterns can also affect the formation of gallstones. A recent cohort study, with an average follow-up of 13.85 years, reported a positive correlation between vegetarians and symptomatic gallstone disease compared with non vegetarians [49]. A case–control study on the relationship between dietary intake and different types of gallstone formation showed that a high consumption of beef, pork and fried food increased the risk of cholesterol stones, while excessive consumption of carbohydrates increased the risk of pigment stones [50].As we did not find the corresponding meta-analysis of the above studies. Therefore, our research does not cover these aspects.

Since the relationship between alcohol consumption and biliary tract diseases is complex and controversial, we conducted a separate study on this topic. First of all, as far as drinking is concerned, studies have confirmed that alcohol is an important risk factor for the occurrence of upper gastrointestinal malignancies. For example, Boffetta et al. [51] reported that acetaldehyde, as the main metabolite of ethanol, may play a role in the occurrence of upper gastrointestinal tumors. While alcohol consumption has been shown to be a risk factor for cancers of the liver, colon and esophagus, it remains controversial when it comes to gallbladder cancer [21]. Of the evidence we reviewed, one suggested that alcohol consumption reduced the risk of gallbladder cancer, one suggested that heavy alcohol consumption increased the risk, and the rest did not suggest an association between alcohol consumption and gallbladder cancer. So far, there is still a lack of high-quality evidence to further clarify the correlation between the two. For cholangiocarcinoma, we did not find moderate or high-grade evidence, but evidence suggests that low alcohol consumption may be a protective factor for cholangiocarcinoma, while heavy alcohol consumption may increase the risk of both intrahepatic and extrahepatic cholangiocarcinoma.

Surprisingly, in terms of cholecystolithiasis/gallbladder diease, regardless of the level of alcohol consumption or the intake of different types of alcoholic beverages, all evidence suggests that drinking is a protective factor for the incidence of cholecystolithiasis/gallbladder diease, although the level of evidence is not high. But overall, given that many studies have reported that drinking is harmful to health, we do not recommend drinking to prevent cholecystolithiasis/gallbladder diease.

### Strengths and limitations

Umbrella reviews is one of the highest level of evidence-based medical evidence at present. It critically evaluates all published meta-analyses and systematic reviews on a medical topic and summarizes evidence from multiple sources [52, 53]. In recent years, the publication of systematic review and meta-analysis research results has increased rapidly. Although this has filled a large number of evidence gaps in clinical decision-making, it also brings difficulties for clinicians in medical decision-making. Therefore, umbrella reviews are becoming increasingly influential in the field of evidence-based medicine.

However, possible limitations should be taken into account in the interpretation of this topic. Firstly, our umbrella review relied only on published systematic reviews and meta-analyses. Some missing individual studies may have had an impact on our results, but the impact was slight because the meta-analyses we included were the most recent, with highest number of studies included. Secondly, for some of the associations we included in this study, the number of original studies included in the corresponding meta-analysis was small, which is likely to result in publication bias. Finally, due to the close correlation between the biliary diseases we studied, and different studies have different classification standards for biliary diseases, we could not achieve a completely unified classification of the diseases in our study, which also affected the research results to some extent.

## Conclusions

Diet and nutrition, as modifiable risk factors, have important implications for prevention, including cancer and other non-communicable diseases. Our study summarizes the current multifaceted evidence on the relationship between dietary and nutritional indicators and biliary diseases. For the prevention of biliary tract diseases, emphasis should be placed on appropriately increasing the intake of fruits, vegetables, coffee and tea, and reducing the intake of alcohol, raw fish and foods with high nitrate. Meanwhile, weight, blood sugar and lipid levels should be controlled, and diabetes should be actively prevented and treated. Drinking is not recommended to prevent gallstones, although studies have shown that it may reduce the risk of cholecystolithiasis. Overall, the quality of all evidence was not high. Evidence from additional high-quality prospective studies are needed in the future.

## Supplementary Information

**Additional file 1: Table S1.** Search terms utilized in the umbrella review.

**Additional file 2: Table S2.** List of excluded studies and exclusion reason.

## Data Availability

Not applicable.
