# Supplementary material for: The role of diet and nutrition related indicators in biliary diseases: an umbrella review of systematic review and meta-analysis
Source: Nutr Metab (Lond). 2022 Jul 30;19:51. doi: 10.1186/s12986-022-00677-1 (PMC9338528; doi:10.1186/s12986-022-00677-1)
Supplement: Supplementary file 1 — Additional file 1: Table S1. Search terms utilized in the umbrella review. [file 12986_2022_677_MOESM1_ESM.pdf]

Supplemental Table S1. Search terms utilized in the umbrella review.(Search date up to 22 November, 2021)

**Datebases searches**(Search date up to 22 November, 2021)					
**Terms 1**	**Terms 2**	**Terms 3**	**Database**	**Restrictions**	**Results**
diet OR dietary OR food OR nutrition OR nutritional factors	“gallbladder cancer” OR “gallbladder carcinoma” OR “gallbladder neoplasms” OR “gallbladder tumor” OR “gallbladder neoplasm” OR “gallbladder mass” OR “gallbladder masses”	meta-analysis OR systematic review OR systematic overview	PUBMED	All field	14
diet OR dietary OR food OR nutrition OR nutritional factors	“biliary Cancer” OR “biliary tumor” OR “biliary neoplasms” OR “biliary neoplasm” OR “biliary mass” OR “biliary masses” OR “cholangiocarcinoma” OR “bile duct cancer” OR “bile duct tumor” OR “bile duct neoplasms” OR “bile duct neoplasm” OR “bile duct mass” OR “bile duct masses”	meta-analysis OR systematic review OR systematic overview	PUBMED	All field	61
diet OR dietary OR food OR nutrition OR nutritional factors	“cholecystolithiasis” OR “gallstone” OR “gallbladder stone”	meta-analysis OR systematic review OR systematic overview	PUBMED	All field	30
TS=(diet OR dietary OR food OR nutrition OR nutritional factors)	TS=(gallbladder cancer OR gallbladder carcinoma OR gallbladder neoplasms OR gallbladder tumor OR gallbladder neoplasm OR gallbladder mass OR gallbladder masses)	TS=(meta-analysis OR systematic review OR systematic overview)	WOS	Topic	52
TS=(diet OR dietary OR food OR nutrition OR nutritional factors)	TS=(biliary Cancer OR biliary tumor OR biliary neoplasms OR biliary neoplasm OR biliary mass OR biliary masses OR cholangiocarcinoma OR bile duct cancer OR bile duct tumor OR bile duct neoplasms OR bile duct neoplasm OR bile duct mass OR bile	TS=(meta-analysis OR systematic review OR systematic overview)	WOS	Topic	77

	duct masses)				
TS=(diet OR dietary OR food OR nutrition OR nutritional factors)	TS=(cholecystolithiasis OR gallstone OR gallbladder stone)	TS=(meta-analysis OR systematic review OR systematic overview)	WOS	Topic	58
diet OR dietary OR food OR nutrition OR nutritional factors	gallbladder cancer OR gallbladder carcinoma OR gallbladder neoplasms OR gallbladder tumor OR gallbladder neoplasm OR gallbladder mass OR gallbladder masses	meta-analysis OR systematic review OR systematic overview	Cochrane	Cochrane Reviews	3
diet OR dietary OR food OR nutrition OR nutritional factors	biliary Cancer OR biliary tumor OR biliary neoplasms OR biliary neoplasm OR biliary mass OR biliary masses OR cholangiocarcinoma OR bile duct cancer OR bile duct tumor OR bile duct neoplasms OR bile duct neoplasm OR bile duct mass OR bile duct masses	meta-analysis OR systematic review OR systematic overview	Cochrane	Cochrane Reviews	7
diet OR dietary OR food OR nutrition OR nutritional factors	cholecystolithiasis OR gallstone OR gallbladder stone	meta-analysis OR systematic review OR systematic overview	Cochrane	Cochrane Reviews	7
Manual searches(Search date up to 22 November, 2021)					
[1] Kyrgiou M, Kalliala I, Markozannes G, et al. Adiposity and cancer at major anatomical sites: umbrella review of the literature. BMJ. 2017. 356: j477.					
[2] Tsilidis KK, Kasimis JC, Lopez DS, Ntzani EE, Ioannidis JP. Type 2 diabetes and cancer: umbrella review of meta-analyses of observational studies. BMJ. 2015. 350: g7607.					
[3] Kim TL, Jeong GH, Yang JW, et al. Tea Consumption and Risk of Cancer: An Umbrella Review and Meta-Analysis of Observational					

	Studies. Adv Nutr. 2020. 11(6): 1437-1452.
[4]	Yi M, Wu X, Zhuang W, et al. Tea Consumption and Health Outcomes: Umbrella Review of Meta-Analyses of Observational Studies in Humans. Mol Nutr Food Res. 2019. 63(16): e1900389.
[5]	Pearson-Stuttard J, Papadimitriou N, Markozannes G, et al. Type 2 Diabetes and Cancer: An Umbrella Review of Observational and Mendelian Randomization Studies. Cancer Epidemiol Biomarkers Prev. 2021. 30(6): 1218-1228.
[6]	Zhu G, Hua J, Wang Z, She F, Chen Y. Tea consumption and risk of gallbladder cancer: A meta-analysis of epidemiological studies. Mol Clin Oncol. 2015. 3(3): 613-618.
[7]	Xiong J, Lin J, Wang A, et al. Tea consumption and the risk of biliary tract cancer: a systematic review and dose-response meta-analysis of observational studies. Oncotarget. 2017. 8(24): 39649-39657.
[8]	Aune D, Vatten LJ, Boffetta P. Tobacco smoking and the risk of gallbladder disease. Eur J Epidemiol. 2016. 31(7): 643-53.
[9]	Gu J, Yan S, Wang B, et al. Type 2 diabetes mellitus and risk of gallbladder cancer: a systematic review and meta-analysis of observational studies. Diabetes Metab Res Rev. 2016. 32(1): 63-72.
[10]	Aune D, Vatten LJ. Diabetes mellitus and the risk of gallbladder disease: A systematic review and meta-analysis of prospective studies. J Diabetes Complications. 2016. 30(2): 368-73.
[11]	Li Y, Yang H, Cao J. Association between alcohol consumption and cancers in the Chinese population--a systematic review and meta-analysis. PLoS One. 2011. 6(4): e18776.
[12]	McGee EE, Jackson SS, Petrick JL, et al. Smoking, Alcohol, and Biliary Tract Cancer Risk: A Pooling Project of 26 Prospective Studies. J Natl Cancer Inst. 2019. 111(12): 1263-1278.
[13]	Ye XH, Huai JP, Ding J, Chen YP, Sun XC. Smoking, alcohol consumption, and the risk of extrahepatic cholangiocarcinoma: a meta-analysis. World J Gastroenterol. 2013. 19(46): 8780-8.
[14]	Wang J, Duan X, Li B, Jiang X. Alcohol consumption and risk of gallstone disease: a meta-analysis. Eur J Gastroenterol Hepatol. 2017. 29(4): e19-e28.
