# Supplementary material for: The role of diet and nutrition related indicators in biliary diseases: an umbrella review of systematic review and meta-analysis
Source: Nutr Metab (Lond). 2022 Jul 30;19:51. doi: 10.1186/s12986-022-00677-1 (PMC9338528; doi:10.1186/s12986-022-00677-1)
Supplement: Supplementary file 2 — Additional file 2: Table S2. List of excluded studies and exclusion reason. [file 12986_2022_677_MOESM2_ESM.pdf]

Supplementary Table S2:List of excluded studies and exclusion reason.

Exclusion reason	Reference number
Title screening excluded:	
Not related to this study.	(1-12)
Not systematic review or meta analysis.	(12-20)
Abstract excluded:	
The subject was not related to biliary cancer or biliary diseases.	(21-23)
The full text cannot be found, and there are newly published studies on the same topic.	(24)
Risk of multiple tumors: There are other more detailed studies on the same subject.	(25-29)
Study on tumor prognosis.	(30)
Full text excluded:	
Study overlap: We chose to include updated and larger sample studies on the same topic.	(31-43)

Reference List

[1] Xue K, Li FF, Chen YW, Zhou YH, He J. Body mass index and the risk of cancer in women compared with men: a meta-analysis of prospective cohort studies. Eur J Cancer Prev. 2017. 26(1): 94-105.

[2] Brenner DR. Cancer incidence due to excess body weight and leisure-time physical inactivity in Canada: implications for prevention. Prev Med. 2014. 66: 131-9.

[3] Burger M, Brönstrup A, Pietrzik K. Derivation of tolerable upper alcohol intake levels in Germany: a systematic review of risks and benefits of moderate alcohol consumption. Prev Med. 2004. 39(1): 111-27.

[4] Arnold M, Renehan AG, Colditz GA. Excess Weight as a Risk Factor Common to Many Cancer Sites: Words of Caution when Interpreting Meta-analytic Evidence. Cancer Epidemiol Biomarkers Prev. 2017. 26(5): 663-665.

[5] Islami F, Goding Sauer A, Gapstur SM, Jemal A. Proportion of Cancer Cases Attributable to Excess Body Weight by US State, 2011-2015. JAMA Oncol. 2019. 5(3): 384-392.

[6] Coe PO, O'Reilly DA, Renehan AG. Excess adiposity and gastrointestinal cancer. Br J Surg. 2014. 101(12): 1518-31; discussion 1531.

[7] Bergström A, Pisani P, Tenet V, Wolk A, Adami HO. Overweight as an avoidable cause of cancer in Europe. Int J Cancer. 2001. 91(3): 421-30.

[8] Rezende L, Lee DH, Louzada M, Song M, Giovannucci E, Eluf-Neto J. Proportion of cancer cases and deaths attributable to lifestyle risk factors in Brazil. Cancer Epidemiol. 2019. 59: 148-157.

[9] Polednak, AP. Estimating the number of US incident cancers attributable to

obesity and the impact on temporal trends in incidence rates for obesity-related cancers. Cancer Detect Prev. 2008. 32(3): 190-199.
[10] Pang Y, Kartsonaki C, Lv J, et al. Observational and Genetic Associations of Body Mass Index and Hepatobiliary Diseases in a Relatively Lean Chinese Population. JAMA Netw Open. 2020. 3(10): e2018721.
[11] Yamin Z, Xuesong B, Guibin Y, Liwei L, Fei L. Risk factors of gallbladder polyps formation in East Asian population: A meta-analysis and systematic review. Asian J Surg. 2020. 43(1): 52-59.
[12] Aune D, Vatten LJ, Boffetta P. Tobacco smoking and the risk of gallbladder disease. Eur J Epidemiol. 2016. 31(7): 643-53.
[13] Kyrgiou M, Kalliala I, Markozannes G, et al. Adiposity and cancer at major anatomical sites: umbrella review of the literature. BMJ. 2017. 356: j477.
[14] Choi EK, Park HB, Lee KH, et al. Body mass index and 20 specific cancers: re-analyses of dose-response meta-analyses of observational studies. Ann Oncol. 2018. 29(3): 749-757.
[15] Alicandro G, Tavani A, La Vecchia C. Coffee and cancer risk: a summary overview. Eur J Cancer Prev. 2017. 26(5): 424-432.
[16] Kotrotsios A, Tasis N, Angelis S, et al. Dietary Intake and Cholelithiasis: A Review. J Long Term Eff Med Implants. 2019. 29(4): 317-326.
[17] Jha G, Kankarla V, McLennon E, et al. Per- and Polyfluoroalkyl Substances (PFAS) in Integrated Crop-Livestock Systems: Environmental Exposure and Human Health Risks. Int J Environ Res Public Health. 2021. 18(23).
[18] Pearson-Stuttard J, Papadimitriou N, Markozannes G, et al. Type 2 Diabetes and Cancer: An Umbrella Review of Observational and Mendelian Randomization Studies. Cancer Epidemiol Biomarkers Prev. 2021. 30(6): 1218-1228.
[19] Tsilidis KK, Kasimis JC, Lopez DS, Ntzani EE, Ioannidis JP. Type 2 diabetes and cancer: umbrella review of meta-analyses of observational studies. BMJ. 2015. 350: g7607.
[20] Kim TL, Jeong GH, Yang JW, et al. Tea Consumption and Risk of Cancer: An Umbrella Review and Meta-Analysis of Observational Studies. Adv Nutr. 2020. 11(6): 1437-1452.
[21] Yi M, Wu X, Zhuang W, et al. Tea Consumption and Health Outcomes: Umbrella Review of Meta-Analyses of Observational Studies in Humans. Mol Nutr Food Res. 2019. 63(16): e1900389.
[22] Worma, N. How high is the Risk of Cancer with moderate Wine Consumption as Part of a Mediterranean Diet? 42ND WORLD CONGRESS OF VINE AND WINE. 2019.
[23] Larsson SC, Wolk A. Coffee consumption and risk of liver cancer: a meta-analysis. Gastroenterology. 2007. 132(5): 1740-5.
[24] Yu C, Cao Q, Chen P, et al. An updated dose-response meta-analysis of coffee consumption and liver cancer risk. Sci Rep. 2016. 6: 37488.
[25] Wang W, Li N. The association of gallstone disease and diabetes mellitus. A meta-analysis. Saudi Med J. 2014. 35(9): 1005-12.

[26] Freisling H, Arnold M, Soerjomataram I, et al. Comparison of general obesity and measures of body fat distribution in older adults in relation to cancer risk: meta-analysis of individual participant data of seven prospective cohorts in Europe. Br J Cancer. 2017. 116(11): 1486-1497.

[27] Freisling, H, Arnold, M, Soerjomataram, I, et.al. Obesity and central obesity in older adults and cancer risk: Meta-analysis of individual participant data from prospective cohort studies of the CHANCES consortium. European Cancer Congress. 2015.

[28] De Ridder J, Julián-Almárcegui C, Mullee A, et al. Comparison of anthropometric measurements of adiposity in relation to cancer risk: a systematic review of prospective studies. Cancer Causes Control. 2016. 27(3): 291-300.

[29] Sacerdote C, Ricceri F. Epidemiological dimensions of the association between type 2 diabetes and cancer: A review of observational studies. Diabetes Res Clin Pract. 2018. 143: 369-377.

[30] Ling S, Brown K, Miksza JK, et al. Risk of cancer incidence and mortality associated with diabetes: A systematic review with trend analysis of 203 cohorts. Nutr Metab Cardiovasc Dis. 2021. 31(1): 14-22.

[31] Lv X, Zhang Z, Yuan W. Pretreatment Prognostic Nutritional Index (PNI) as a Prognostic Factor in Patients with Biliary Tract Cancer: A Meta-Analysis. Nutr Cancer. 2021. 73(10): 1872-1881.

[32] Tan W, Gao M, Liu N, Zhang G, Xu T, Cui W. Body Mass Index and Risk of Gallbladder Cancer: Systematic Review and Meta-Analysis of Observational Studies. Nutrients. 2015. 7(10): 8321-34.

[33] Larsson SC, Wolk A. Obesity and the risk of gallbladder cancer: a meta-analysis. Br J Cancer. 2007. 96(9): 1457-61.

[34] Martínez de Pancorbo C, Carballo F, Horcajo P, et al. Prevalence and associated factors for gallstone disease: results of a population survey in Spain. J Clin Epidemiol. 1997. 50(12): 1347-55.

[35] Wang J, Duan X, Li B, Jiang X. Alcohol consumption and risk of gallstone disease: a meta-analysis. Eur J Gastroenterol Hepatol. 2017. 29(4): e19-e28.

[36] Palmer WC, Patel T. Are common factors involved in the pathogenesis of primary liver cancers? A meta-analysis of risk factors for intrahepatic cholangiocarcinoma. J Hepatol. 2012. 57(1): 69-76.

[37] Songserm N, Woradet S, Charoenbut P. Fruit and Vegetables Consumption: A Pointer for Cholangiocarcinoma Prevention in Northeast Thailand, the Highest Incidence Area in the World. Nutr Cancer. 2016. 68(8): 1289-1294.

[38] Kan HP, Huang YQ, Tan YF, Zhou J. Meta-analysis of alcohol consumption and risk of extrahepatic bile system cancer. Hepatol Res. 2011. 41(8): 746-53.

[39] Li JS, Han TJ, Jing N, et al. Obesity and the risk of cholangiocarcinoma: a meta-analysis. Tumour Biol. 2014. 35(7): 6831-8.

[40] Chen Y, Wu F, Saito E, et al. Association between type 2 diabetes and risk of cancer mortality: a pooled analysis of over 771,000 individuals in the Asia Cohort Consortium. Diabetologia. 2017. 60(6): 1022-1032.

[41] Park M, Song DY, Je Y, Lee JE. Body mass index and biliary tract disease: a systematic review and meta-analysis of prospective studies. Prev Med. 2014. 65: 13-22.
[42] Renehan AG, Tyson M, Egger M, Heller RF, Zwahlen M. Body-mass index and incidence of cancer: a systematic review and meta-analysis of prospective observational studies. Lancet. 2008. 371(9612): 569-78.
[43] Body Size Indicators and Risk of Gallbladder Cancer: Pooled Analysis of Individual-Level Data from 19 Prospective Cohort Studies[J]. Cancer epidemiology, biomarkers and prevention: A publication of the American Association for Cancer Research, 2017, 26(4):597-606.
[44] Li ZM, Wu ZX, Han B, et al. The association between BMI and gallbladder cancer risk: a meta-analysis. Oncotarget. 2016. 7(28): 43669-43679.
